# Bacterial Communities in Boreal Forest Mushrooms Are Shaped Both by Soil Parameters and Host Identity

**DOI:** 10.3389/fmicb.2017.00836

**Published:** 2017-05-10

**Authors:** Mari Pent, Kadri Põldmaa, Mohammad Bahram

**Affiliations:** ^1^Department of Botany, Institute of Ecology and Earth Sciences, University of TartuTartu, Estonia; ^2^Department of Organismal Biology, Evolutionary Biology Centre, Uppsala UniversityUppsala, Sweden

**Keywords:** Agaricales, Boletales, ectomycorrhizal fungi, food microbiome, microbial interactions, Proteobacteria, Russulales, symbiont communities

## Abstract

Despite recent advances in understanding the microbiome of eukaryotes, little is known about microbial communities in fungi. Here we investigate the structure of bacterial communities in mushrooms, including common edible ones, with respect to biotic and abiotic factors in the boreal forest. Using a combination of culture-based and Illumina high-throughput sequencing, we characterized the bacterial communities in fruitbodies of fungi from eight genera spanning four orders of the class Agaricomycetes (Basidiomycota). Our results revealed that soil pH followed by fungal identity are the main determinants of the structure of bacterial communities in mushrooms. While almost half of fruitbody bacteria were also detected from soil, the abundance of several bacterial taxa differed considerably between the two environments. The effect of host identity was significant at the fungal genus and order level and could to some extent be ascribed to the distinct bacterial community of the chanterelle, representing Cantharellales—the earliest diverged group of mushroom-forming basidiomycetes. These data suggest that besides the substantial contribution of soil as a major taxa source of bacterial communities in mushrooms, the structure of these communities is also affected by the identity of the host. Thus, bacteria inhabiting fungal fruitbodies may be non-randomly selected from environment based on their symbiotic functions and/or habitat requirements.

## Introduction

Bacteria are ubiquitous microbes in many host and non-host environments, where they play essential roles in nutrient cycling. In eukaryotic hosts, bacteria may also perform various pathogenic and mutualistic functions, such as improving nutrient uptake, growth, and protection of their hosts against pathogens (Eckburg et al., 2006; Grice et al., 2009; Hacquard and Schadt, 2014; Vandenkoornhuyse et al., 2015). Besides their ecological importance, bacteria colonizing edible plants or animals may also impact conservation and healthfulness of derived food products. For example, several probiotic and bacteria antagonistic to human pathogens have been found in fresh fruits, vegetables and truffles (Trias et al., 2008; Vitali et al., 2012; Saidi et al., 2015). Despite the increasing knowledge of the microbiome in eukaryotes (Bäckhed et al., 2005; Grube et al., 2009; Fan et al., 2012; Bulgarelli et al., 2013; Hyde et al., 2016), many major host groups remain little studied in this regard.

Fungi represent a highly diverse group of eukaryotes occurring in most ecosystems. The most conspicuous fungi include mushrooms with aboveground spore-forming structures (fruitbodies) in various groups of Basidiomycota and certain Ascomycota. Some members of these two phyla are economically important sources of food and medicine for humans. Although mushrooms have one of the largest fruitbodies among fungi, potentially harboring highly diverse bacteria, evidence of their microbiome is limited and almost exclusively to taxa that grow in pure culture (Rangel-Castro et al., 2002a; Tsukamoto et al., 2002; Kumari et al., 2013). Only a few studies have analyzed mushrooms from different genera in this regard (Zagriadskaia et al., 2013; de Carvalho et al., 2015). More comprehensive studies on characterizing the bacterial communities using next generation sequencing methods have focused of truffles and similar ascomycetes with belowground fruitbodies (e.g., Antony-Babu et al., 2014; Benucci and Bonito, 2016). Bacteria may have several symbiotic functions in mushrooms, such as inhibiting pathogens and antagonists (Tsukamoto et al., 2002; Frey-Klett et al., 2007), improving the distribution of spores (Citterio et al., 2001; Splivallo et al., 2014) or providing vitamins and growth regulators (Rangel-Castro et al., 2002a; Riedlinger et al., 2006). Several fungal-associated bacteria are also known to fix nitrogen (Jayasinghearachchi and Seneviratne, 2004; Paul et al., 2007; Barbieri et al., 2010; Hoppe et al., 2014), although there is yet no evidence that the fungus directly benefits from that ability of the associated bacteria. Increasing evidence shows that fruitbody formation in mushrooms can be triggered (Noble et al., 2009) or inhibited (Munsch et al., 2002; Yun et al., 2013) by bacteria. The exploration of fungal microbiome can thus be useful for improving the yield of cultivated mushrooms (Cho et al., 2003; Barbieri et al., 2010; Kataoka et al., 2012) and for identifying bacteria antagonistic to fungal pathogens (De Boer et al., 2005; Bandara et al., 2006).

Similar to the pattern observed in several eukaryotes that share their microbiota with that of the surrounding environment (Fan et al., 2012; Jeon et al., 2013), a large proportion of bacteria in mushrooms could be adopted from their environment. Majority of the lifecycle of mushrooms occurs in the form of mycelia in their substrata, mainly soil, whereas fruitbodies are mostly formed above the ground. Soil contains diverse bacterial communities (Fierer and Jackson, 2006; Rousk et al., 2010), that provide a species pool for the microbiomes of soil-inhabiting organisms (Antony-Babu et al., 2014; Deveau et al., 2016). Thus, environmental forces that shape bacterial community composition in soil may indirectly contribute to the structure of bacterial communities in fungal mycelia and fruitbodies (Warmink et al., 2009; Antony-Babu et al., 2014). In case of mushrooms, acquisition of bacteria from air has also been suggested (Zagriadskaia et al., 2013). In addition to abiotic factors, host identity has been observed to affect the community composition of mycorrhiza-helper bacteria (Poole et al., 2001; Frey-Klett et al., 2007) as well as that of bacteria in the mycosphere (Warmink et al., 2009) and fungal fruitbodies (Dahm et al., 2005; Kumari et al., 2013). In particular, fruitbodies of different fungal taxa create various specific conditions that filter certain bacteria from the surrounding bulk soil and the mycosphere (Boersma et al., 2009, 2010; Antony-Babu et al., 2014). Such selectivity has been observed in plants, which select bacteria present in soil depending on host’s genotype (Vandenkoornhuyse et al., 2015), rhizodeposits (Bulgarelli et al., 2013), and several niche-specific factors such as temperature, pH, oxygen levels, and organic carbon availability (Bulgarelli et al., 2013; Hacquard et al., 2015).

In this study, we aimed to characterize the bacterial communities of mushrooms in boreal forests and to understand the factors shaping their structure. In particular, our main goal was to examine the impact of fungal identity, habitat, and soil type as well as soil parameters on the bacterial community composition in fungal fruitbodies. We hypothesized that these communities originate from underlying soils, hence the bacterial community structure in mushrooms depends mainly on soil characteristics. To test this hypothesis, we compared the bacterial communities in fruitbodies of ectomycorrhizal (EcM) fungi from four main mushroom-forming fungal orders as well as in the adjacent soil, using a combination of culture-based and high-throughput sequencing (HTS).

## Materials and Methods

### Study Sites and Fruitbody Sampling

Fungal fruitbodies were collected in boreal forests at three nature reserves in Eastern Estonia in September–October 2014 and 2015. Altogether four 2,500 m^2^ plots, located in three nature reserves, were sampled from each of the three habitat types (Supplementary Table S1). Mushrooms were identified to 15 EcM species (except for unidentified *Russula* spp.) from the four main orders of mushroom-forming fungi (Basidiomycota). The number of fruitbodies collected depended on their presence at each site; in case no fruitbodies were found for a particular species, fruitbodies of congeneric species were sampled (**Table 1**). Only mature fruitbodies were sampled, excluding immature and decaying mushrooms from our selection. All fruitbodies were packed individually in foil, transported to the lab in a cooled container and kept in fridge at 4°C until being handled in a laminar flow chamber.

**Table 1:** Mushrooms taxonomic identity and number of fruitbodies sampled at each site.

Order	Family	Genus	Species	Number of analyzed fruitbodies		Number of sites	
					
				HTS^a^	Culture	HTS	Culture
Agaricales	Amanitaceae	*Amanita*	*A. fulva*	18	23	7	6
Agaricales	Amanitaceae	*Amanita*	*A. muscaria*	0	1	0	1
Agaricales	Amanitaceae	*Amanita*	*A. rubescens*	0	1	0	1
Agaricales	Cortinariaceae	*Cortinarius*	*C. caperatus*	14	6	4	3
Agaricales	Cortinariaceae	*Cortinarius*	*C. armillatus*	10	9	4	4
Cantharellales	Cantharellaceae	*Cantharellus*	*C. cibarius*	12	12	5	5
Boletales	Boletaceae	*Leccinum*	*L. holopus*	7 (2)	7	3	3
Boletales	Boletaceae	*Leccinum*	*L. scabrum*	8	4	3	3
Boletales	Boletaceae	*Leccinum*	*L. variicolor*	6	3	2	2
Boletales	Paxillaceae	*Paxillus*	*P. involutus*	9	5	3	2
Boletales	Suillaceae	*Suillus*	*S. bovinus*	17	15	5	4
Boletales	Suillaceae	*Suillus*	*S. variegatus*	22 (1)	18	9	7
Russulales	Russulaceae	*Russula*	*R. decolorans*	11	13	4	5
Russulales	Russulaceae	*Russula*	*Russula* spp.^b^	21	19	7	8
Russulales	Russulaceae	*Lactarius*	*L. quieticolor*	3	3	1	1
Russulales	Russulaceae	*Lactarius*	*L. rufus*	25	38	8	9


*^a^Number of fruitbodies with no HTS reads of bacterial taxa are given in parentheses. ^b^Including *Russula emetica*, *Russula paludosa*, *Russula rhodopus*, and *Russula vinosa*.*

### Soil Characteristics

Soil samples originated from the same sites as the mushrooms and were collected according to the methodology described by Tedersoo et al. (2014). Briefly, two soil samples were collected near the base of each of the 20 trees growing at nearly equal distance from each other and the 40 soil samples from a site were pooled for all subsequent analyses. The fruitbodies were collected from close proximity to the selected trees, if possible. The concentrations of organic matter, carbon (C), nitrogen (N), δ^15^N, phosphorus (P), potassium (K), calcium (Ca), and magnesium (Mg) were found as described by Tedersoo et al. (2012). δ^15^N was included in our analysis as a measure of nitrogen availability. Soil pH was measured in 1 M KCl solution. Soil types and parameters are listed in Supplementary Table S2.

### Culturing

To avoid contaminations, only intact tissue from inside mushrooms was allocated for analyses. For that purpose, fruitbody samples were cut lengthwise using a sterile scalpel, followed by sterilizing the cut surface of the two halves under the UV light for 5 min to avoid cross contamination. The second round of cuts was made along the cut surface of the cap, the central part and along the lower part of the stipe. Care was taken to avoid contact with the surface of the fruitbody. The scalpel was flame-sterilized before every single cut. Using a sterile drill, two 5 mm^3^ pieces of fungal tissue were taken from each of the three double-cut areas at the longitudinal section.

Two sets of three pieces (one from the cap and one from the middle part and one from the lower part of the stipe) from each fruitbody were separated into two 1.5 ml Eppendorf tubes, one of which was kept at -20°C for HTS. In the other, containing 400 μl of 0.1 M phosphate buffer (1 M SmartMix, pH 7, Naxo OÜ, Estonia), fruitbody pieces were crushed with a sterile scalpel and vortexed for 5 min at maximum speed to isolate tightly adhering bacteria from the hyphal surface. Using a Drigalski spatula, 100 μl of the homogenate was plated to one Petri dish with R2A low nutrient agar or in some cases onto twice diluted tryptic soybean agar (TSA, Liofilchem, Italy). Both media have successfully been used for isolation of bacteria from fungal fruitbodies or from lichen thalli (e.g., Dahm et al., 2005; Grube et al., 2009). The plates were incubated at 25°C for 30 days not to miss the slow-growing bacteria. From each Petri dish, colonies with a different size, shape, elevation, color, margin, texture, surface, or opacity were transferred to a new Petri dish with TSA. Most of the isolates were examined using the light microscope to record their Gram reaction and cell shape. Reinoculation was repeated until pure isolates were obtained. Cultures were preserved at -80°C in 50% glycerol in the Tartu Fungal Culture Collection (TFC).

### DNA Extraction

#### Culture Strains

Bacteria from 2–4 days old colonies of each isolate on TSA were transferred into 100 μl of lysis buffer containing 0.8 M Tris–HCl, 0.2 M (NH_4_)_2_SO_4_, 0.2% w/v Tween-20 (10× Reaction Buffer B, Solis Biodyne, Tartu, Estonia), and 2.5 μl of proteinase K (20 mg/ml, Fermentas, Lithuania). The probes were incubated at 56°C for 15–16 h, followed by incubation at 98°C for 15 min to inactivate proteinase K. From each probe, 16S rRNA gene was amplified using the universal bacterial primers 27f and 1492r under the following PCR conditions: 95°C for 15 min, 30 cycles of 95°C for 30 s, 58°C for 30 s, and 72°C for 1 min and the final elongation step at 72°C for 10 min. The 25 μl PCR mix consisted of 5 μl of 5× HOT FIREPol Blend MasterMix (Solis Biodyne, Tartu, Estonia), 1 μl of 10-fold diluted DNA extract, 0.5 μl of each primer (200 nM) and 18 μl of sterilized H_2_O. The PCR products were visualized on 1% agarose gel and purified using ExoSAP-IT (USB Corporation, Cleveland, OH, USA). Purified PCR products were sequenced using the Sanger method at the Macrogen Inc. (Amsterdam). Sequences obtained from all isolates have been uploaded to GenBank, with accession numbers KY681818–KY682069 (Supplementary Table S3).

#### High-Throughput Sequencing

The frozen fruitbody pieces were crushed for 3 min in 200 μl of phosphate-buffered saline water solution (PBS) (0.15 M NaCl 10 mM phosphate buffer, pH 7.4) using 3.2 mm diameter metal beads followed by centrifuging the homogenate at 3,000 × *g* for 30 s. DNA was extracted from the supernatant using the High Pure PCR Template Preparation Kit (Roche Applied Science, Mannheim, Germany) following the manufacturer’s instructions for isolation of bacteria. PowerMax Soil DNA Isolation Kit (MoBio, Carlsbad, CA, USA) was used for the extraction of DNA from soil following the manufacturer’s instructions.

The variable V3–V4 regions of 16S rDNA gene were amplified using the bacterial primers 515F (5′-GTGYCAGCMGCCGCGGTAA-3′) and 806R (5′-GGACTACNVGGGTWTCTAAT-3′). The 25 μl of PCR mix consisted of 16 μl of sterilized H_2_O, 5 μl of 5× HOT FIREPol Blend MasterMix (Solis Biodyne, Tartu, Estonia), 0.5 μl of each primer (200 nM), and 3 μl of the DNA extract. Amplifications were performed using the following PCR conditions: 95°C for 15 min, followed by 25–30 cycles of 95°C for 30 s, 50°C 45 s, and 72°C for 1 min with a final extension step at 72°C for 10 min. The PCR products were visualized on 1% agarose gel. For DNA samples in which no PCR product could be amplified, up to 35 PCR cycles were applied (Supplementary Table S4). The PCR products were sequenced at the Estonian Biocentre (Tartu, Estonia) using Illumina MiSeq technology. Representative sequence from each operational taxonomic unit (OTU) was submitted to GenBank and can be retrieved from BioProject PRJNA379722: Fungal bacteria.

### Sequence Analysis

The sequences obtained from culture isolates were assembled and manually edited using *Sequencher 5.1* (Gene Codes Corporation, USA) and subsequently aligned using MAFFT^1^. The program AliView (Larsson, 2014) was used to trim the nearly full-length 16S rRNA sequences to correspond to the positions 56–1,461 in *E. coli* or to positions 323–1,461 in case only the reverse primer was used for sequencing. Sequence similarity searches were performed using SILVA^2^, nucleotide-nucleotide basic local alignment search tool (BLASTn) in GenBank and the GreenGenes database^3^.

Illumina sequences from soil and fungal samples, 906,778 raw reads in total, were processed using the software package LotuS (Hildebrand et al., 2014). The primer and barcode region were removed from each read and sequences shorter than 170 nucleotides or those detected as chimeric excluded. The remaining reads, 247 bp in length, were clustered into OTUs with UPARSE based on 97% sequence similarity threshold. OTUs that occurred in high numbers in negative controls (52,325 reads) and those with affinities in eukaryotes (250,268 reads), Archaea (79 reads), or of unknown origin (152,631 reads), accounting for 57% of quality-filtered sequences, were discarded, resulting in 247,125 quality-passed reads from 955 bacterial OTUs, on average 1,765 reads per sample. Seventy-six OTUs (754 reads), each represented only in one sample, as well as OTUs with less than five reads in total, were excluded from downstream analyses. Representative sequences from each bacterial OTU were classified using the SILVA database and compared with most similar sequences in GenBank, RDP^4^ and in GreenGenes databases. The final taxonomy was determined based on the best blast match for a given representative sequence in any of the above databases. Their affinities at the species, genus, family, order, class, and phylum level were assigned applying the sequence similarity thresholds of 97, 94.5, 86.5, 82.0, 78.5, and 75.0%, respectively (Yarza et al., 2014).

For OTU-level comparison of HTS-detected bacteria with those isolated into culture, the V3–V4 regions were extracted from the full-length 16S rDNA sequences from the latter using the program AliView. These spanned from positions 534 to 781 in *E. coli*, corresponding to the same 247 bp stretch obtained with HTS and were clustered with quality-filtered Illumina sequences using cd-hit-est at 97% sequence similarity in the program CD-HIT (Huang et al., 2010). For analyzing together HTS and Sanger sequencing data from fruitbodies, these from each fungal species at a particular site were pooled and transformed into the presence/absence form. The relative abundance of bacterial taxa in fruitbodies was calculated separately in case of the datasets obtained from Sanger sequencing of culture isolates and from HTS of fungal tissues. For each fungal taxon, the abundance of individual bacterial groups was calculated as the proportion of infested fruitbodies among sampled ones or as the relative proportion of sequence reads in a particular fungal taxon, respectively. For calculating the relative abundance of bacterial taxa in soil, the read number of each bacterial taxon was divided by the total number of reads detected from the 12 samples. The soil data was not rarefied as it was not included in any of the statistical analyses conducted only with fruitbody data.

### Statistical Analysis

In the final HTS based bacterial OTU community table, sequence counts of all fruitbodies of one fungal species at one site were merged into one sample. Three samples with <12 sequences were excluded from statistical analyses, leaving 64 for downstream analyses. Because the total read numbers differed substantially, 49 samples with > 100 reads were rarefied to 100 reads per sample and merged with rest of the samples. The resulting OTU table was further normalized using Hellinger transformation, and OTUs represented by a single sequence (singletons) were excluded from data analysis. The vegan package in R (vers.3.2.2, R Development Inc., 2013) was used for all these procedures.

Permutational analysis of variation (PERMANOVA) was performed using Adonis function to determine the effect of sample site, habitat type, soil type, fungal host identity, and soil parameters on bacterial community composition in fungal fruitbodies. The best model was selected based on forward selection with *F*-value as the selection criterion. Canonical analysis of principal (CAP) coordinates, based on Bray–Curtis dissimilarity (Anderson and Willis, 2003), was applied to visualize differences in bacterial communities among fungal taxa at three taxonomic levels (order, genus, species) and to visualize the effect of soil parameters underlying the observed variation based on Illumina data. CAP was performed using the program Primer 6 (Primer-E Ltd., Plymouth, UK) with the PERMANOVA+ add-on package. The function betadisper in the vegan package was used for analysis of homogeneity of groups dispersions followed by Tukey’s honestly significant difference (HSD) tests. The program EstimateS 9.1.0 (Colwell et al., 2012) was used for finding the estimators for Coleman rarefaction curves.

## Results

### Structure of Bacterial Communities in Mushrooms

**FIGURE 1:**
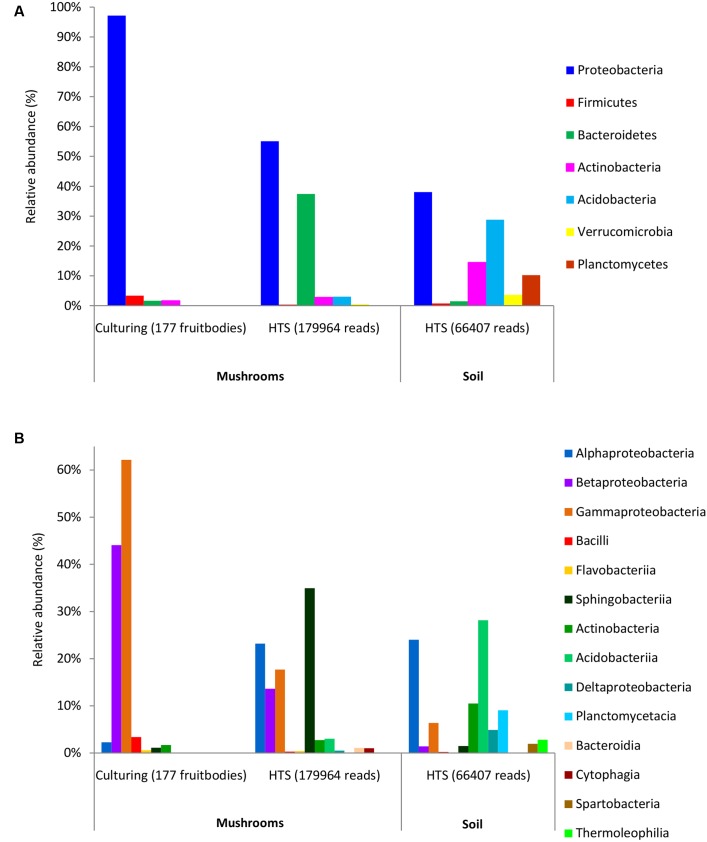
**Relative abundance of dominant bacterial phyla** **(A)** and classes **(B)** in mushrooms based on culturing and high-throughput sequencing (HTS), and in soil based on HTS. The ratios show the share of fruitbodies inhabited by the bacterial taxon or of Illumina read numbers from that of the total (in parentheses) for culture isolates and HTS data, respectively.

Bacterial colonies were successfully isolated from 177 (out of 221) fruitbodies used for inoculation on agar media. In total, 252 bacterial strains were isolated after reinoculation of different colonies from each original Petri dish (Supplementary Table S3). Most of these bacteria were Gram-negative rods, except for two Gram-positive cocci (*Staphylococcus pasteuri* and *Staphylococcus epidermidis*), and two Gram-positive rods (*Microbacterium aurum* and *Frondicola australicus*). Based on full-length 16S rDNA sequences, the isolated bacteria were identified as belonging to 4 phyla, 7 classes, 10 orders, 13 families, 17 genera, and 54 species, whereas no names could be assigned to some isolates at the genus or species level (Supplementary Table S3). Clustering that applied 97 or 98.6% similarity threshold revealed 37 and 58 species-level groups, respectively. The phylum Proteobacteria (present in 97% of fruitbodies) and its two classes, Gammaproteobacteria and Betaproteobacteria, were the dominant taxa among the isolates (Supplementary Table S5 and **Figures 1A,B**). At lower taxonomic levels, the families Burkholderiaceae (found in 42% of fruitbodies), Pseudomonadaceae (36%), Enterobacteriaceae (33%), and respective orders were most common. The most abundant genera, *Pseudomonas* and *Burkholderia*, included *Pseudomonas fluorescens* and *Pseudomonas brenneri* (both in 14% of fruitbodies); *Burkholderia phytofirmans* (in 10%), *Burkholderia phenazinium* (8%), *Burkholderia xenovorans* (6%), *Burkholderia bryophila* (6%), and *Burkholderia graminis* (5%). Other bacterial taxa, at all six taxonomic levels, grew out in culture from less than 5% of fruitbodies. With respect to host taxa, different bacterial groups often dominated among isolates obtained from different fungal genera (Supplementary Table S5). For example, *Pseudomonas* was the most common bacterial genus isolated from *Cantharellus* and *Amanita* but least frequent in the three genera of Boletaceae, from which Enterobacteriaceae/-les were most frequently isolated.

**FIGURE 2:**
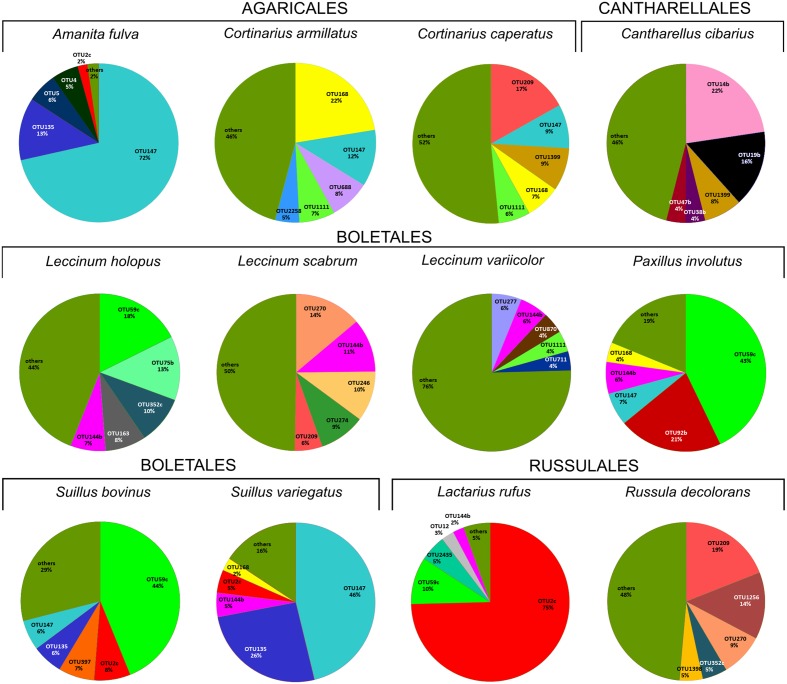
**Relative abundance of five dominant bacterial OTUs in each studied fungal species based on HTS data.** The section “others” presents the proportion of all the remaining, less numerous OTUs. The OTUs were assigned to following taxa: OTU209 *Enhydrobacter* sp., OTU14b *Chitinophaga* sp., OTU38b unclassified Chitinophagaceae, OTU144b *Bacteroides* sp., OTU168 *Cytophaga* sp., OTU47b *Pedobacter cryoconitis*, OTU688 unclassified Cytophagales, OTU1111 *Sphingomonas* sp., OTU2258 *Curtobacterium* sp., OTU277 unclassified Rhizobiales, OTU19b *Rhizobium* sp., OTU870 unclassified Rhizobiales, OTU711 *Planctomyces* sp., OTU270 *Chryseobacterium* sp., OTU246 unclassified Caulobacteraceae, OTU59c *Burkholderia* sp., OTU274 *Brevundimonas* sp., OTU135 unclassified Enterobacteriaceae, OTU5 *Janthinobacterium lividum*, OTU4 *Pseudomonas* sp., OTU2c *Burkholderia* sp., OTU75b *Novosphingobium* sp., OTU352c *Massilia* sp., OTU163 *Luteibacter rhizovicinus*, OTU2435 *Dyella* sp., OTU12 *Shewanella algae*, OTU9 *Mucilaginibacter* sp., OTU58b *Telmatospirillum* sp., OTU1043 *Acinetobacter lwoffii*, OTU4226 *Pseudonocardia* sp., OTU1256 *Paracoccus* sp., OTU92b unclassified Alphaproteobacteria, OTU397 *Corynebacterium* sp., OTU147 *Pseudomonas* sp., OTU1399 *Pedobacter* sp., OTU1398 *Wolbachia* sp.

HTS detected 179,964 bacterial reads from 178 mushroom fruitbodies, on average 2,812 reads per the 64 pooled samples. These sequences clustered into 446 bacterial OTUs that belonged to 24 (including five unclassified) bacterial phyla, 44 (11 unclassified) classes, 99 (32 unclassified) orders, 157 (53 unclassified) families, and 306 (153 unclassified) genera (Supplementary Tables S6, S7). Proteobacteria was the most abundant phylum in all fungal genera, except for *Cantharellus*, where Bacteroidetes was of equal abundance (Supplementary Table S5 and Figure S1). The identity and relative abundance of the five main bacterial OTUs was considerably different among mushroom species (**Figure 2**). A *Pseudomonas* (OTU 147) was common in all three Agaricales and three boletes, whereas two species of *Burkholderia*, OTUs 59c and 2c, dominated in three boletes or in *Lactarius rufus*, respectively. *Cortinarius caperatus* and *Russula decolorans* shared their dominant, *Enhydrobacter* sp. (OTU 209), whereas *Cantharellus cibarius*, with the most distinct bacterial community, had only *Pedobacter* sp. (OTU 1399) in common with *C. caperatus*.

The 97% similarity-based clustering of V3–V4 regions of 16S sequences obtained from isolates distinguished 25 OTUs, eight of which were not detected with HTS. Although infrequent, two of such OTUs represented also unique genera (*Plantibacter* and *Flavobacterium*) in our dataset. All bacterial families and higher level taxa observed in culture were also detected by HTS. However, Enterobacteriales, Pseudomonadales, and Burkholderiales were poorly represented among HTS reads, yet isolated from over one third of the fruitbodies (Supplementary Table S5). By contrast, Alphaproteobacteria and Sphingobacteriia/Bacteroidetes were abundant in the HTS dataset (**Figures 1A,B**) but rare or undetected in culture.

### Comparison of Bacterial Communities in Fungal Fruitbodies and in Soil

**FIGURE 3:**
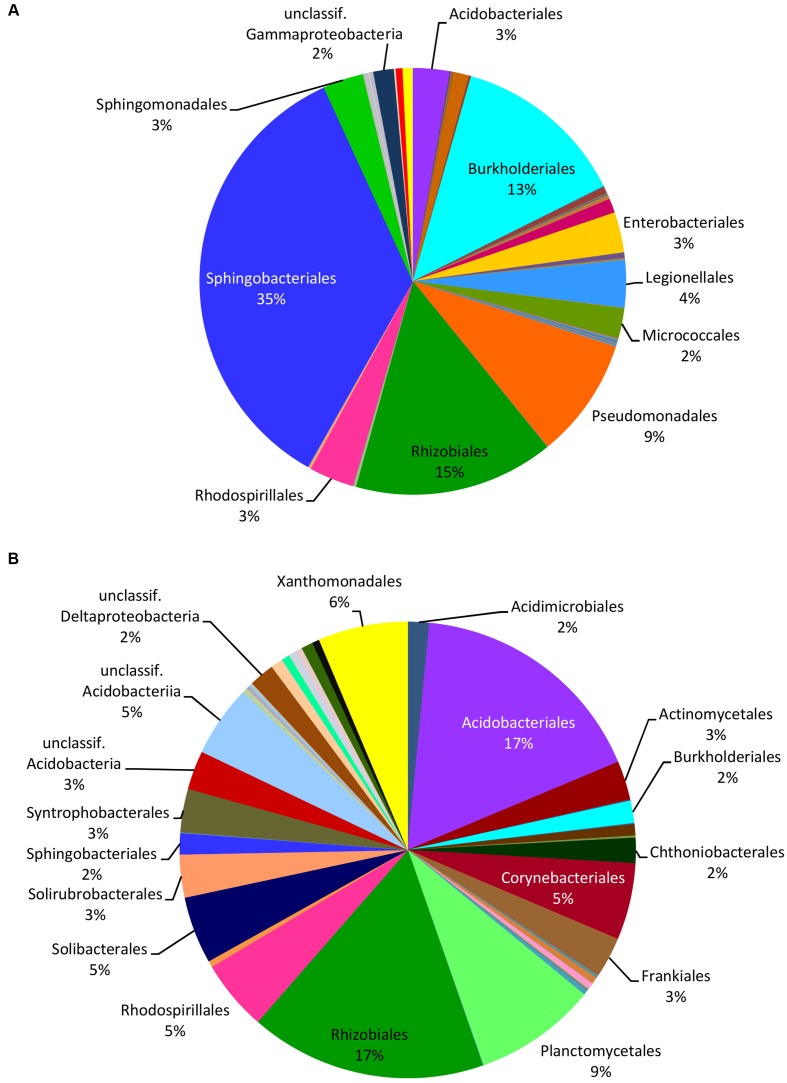
**Relative distribution of the bacterial orders in mushrooms** **(A)** and in soil **(B)** based on the read numbers normalized by the total number of reads from HTS data. Names of the taxa are presented only for bacterial groups with abundance ≥2%.

HTS from the 12 soil samples detected 66,407 quality-filtered reads that clustered into 639 OTUs belonging to 24 (including five unclassified) bacterial phyla, 51 (21 unclassified) classes, 93 (43 unclassified) orders, 160 (90 unclassified) families, and 406 (314 unclassified) genera (Supplementary Table S8). Rarefaction curves showed that the number of bacterial OTUs with increasing number of reads reached a plateau in case of mushrooms but not in soil bacteria (Supplementary Figure S2). In general, 21.6% of all bacterial OTUs detected by HTS from mushrooms and soil were shared (Supplementary Figure S3), while 31% of the OTUs in soil also occurred in fruitbodies and 41% of fruitbody OTUs were present in soil. Although most of the commonly observed bacterial taxa occurred both in mushrooms and in soil, their relative abundance often differed significantly between these two habitats. Namely, Proteobacteria was the most abundant phylum both in fruitbodies and in soil, whereas it was followed by Bacteroidetes in fruitbodies, but Acidobacteria and Actinobacteria in soil samples (**Figure 1A**). At the class level, Acidobacteriia was most abundant in soil and Sphingobacteriia in mushrooms, followed by Alphaproteobacteria in both (**Figure 1B** and Supplementary Figure S4). The structure of bacterial communities in these two environments differed also at the bacterial order level (**Figure 3**). Bacterial taxa detected only from mushrooms included three phyla (Candidatus Saccharibacteria, Deinococcus–Thermus, and Candidate division OP3), eight classes and 24 orders (Supplementary Tables S6, S7). By contrast, three phyla, two classes, and 18 orders were found only from soil (Supplementary Tables S6, S8).

### Factors Affecting the Structure of Mushroom-Associated Bacterial Communities

**FIGURE 4:**
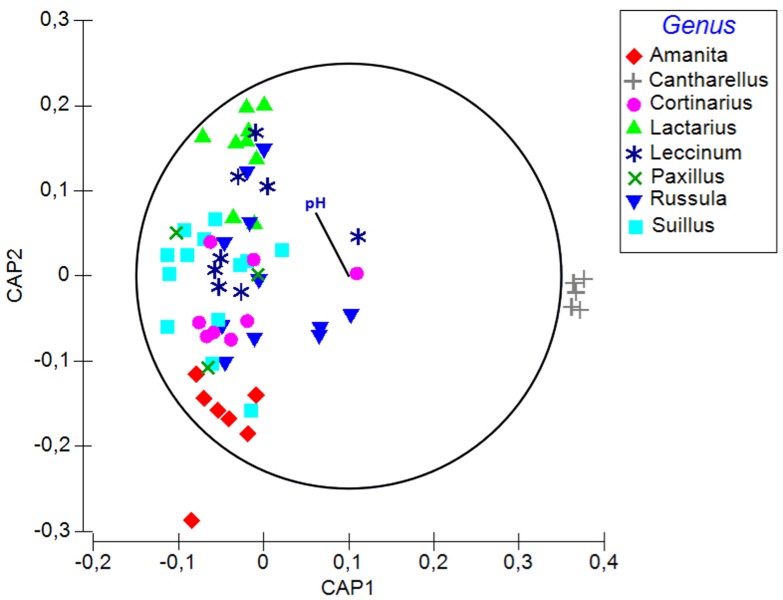
**A plot of the canonical analysis of principal (CAP) coordinates visualizing the differences in bacterial communities among fungal genera and the effect of soil parameters underlying the observed variation based on Bray–Curtis dissimilarity of HTS data.** Vector shows Pearson correlations with soil pH along the second axis of CAP (variables with correlations ≥0.3 presented). Correlation coefficient for pH is –0.26.

Soil pH, δ15N, fungal order, and genus were the best determinants of the bacterial community composition in fungal fruitbodies based on PERMANOVA analysis (**Table 2** and Supplementary Table S9). Similar results were obtained when using the presence/absence matrix of merged culture and HTS data (Supplementary Table S10). The effect of host identity and soil pH appeared to be independent (**Table 2** and Supplementary Table S11). CAP analysis separated the four fungal orders (Supplementary Figure S5), eight genera (**Figure 4**), and 14 species (Supplementary Figure S6) with respect to their bacterial communities along the second axis of CAP, mainly correlated to pH and some other soil variables. The CAP plot revealed lower intraspecific variation of the distinct bacterial communities in *C. cibarius* compared to that in other mushroom species with largely overlapping bacterial community composition (Supplementary Figure S6). The same pattern was distinguished at higher fungal taxonomic levels, with bacterial communities of *Cantharellus* and Cantharellales being most distinct from those of other fungal taxa. The Tukey’s test confirmed the distinctness of bacterial communities in Cantharellales by revealing significant differences (*p* < 0.05) in bacterial community composition between all pairs of orders that involved Cantharellales (C): C-Agaricales, C-Boletales and C-Russulales (Supplementary Figure S7).

**Table 2:** Effect of soil variables and host identity on bacterial community composition in fungal fruitbodies as revealed by PERMANOVA of rarefied, Hellinger transformed HTS read numbers.

	Df	SS	MS	*F*	*R*^2^	*R*^2^ adjusted	*p*
Soil pH	1	1.9065	1.90646	6.2427	0.07690	0.0620	0.001
Fungal order	3	3.3701	1.12338	3.6785	0.13594	0.0927	0.001
Soil δ15N	1	0.7247	0.72472	2.3731	0.02923	0.0136	0.009
Fungal genus	4	2.2984	0.57461	1.8816	0.09271	0.0312	0.002
Residuals	54	16.4910	0.30539		0.66521	-1.34353	
Total	63	24.7907			1	1	


**p*-value is based on 999 permutations.*

## Discussion

### Determinants of Bacterial Communities in Mushrooms

This study presents the first comprehensive analysis of the structure of bacterial communities in forest mushrooms. We found that bacterial communities across the eight studied mushroom genera were primarily affected by soil pH—the main determinant of bacterial diversity and community composition in soil (Fierer and Jackson, 2006; Lauber et al., 2009; Rousk et al., 2010). This effect was likely mediated by the indirect impact of soil pH on surrounding soil bacterial communities that appeared to provide a substantial part of bacteria in mushrooms. Although Proteobacteria dominated on both habitats, contrasting structure of bacterial communities was observed in soil and fungal fruitbodies, from which 41% of OTUs were also detected from adjacent soil samples. However, part of these community differences probably resulted from the different time and methodology used for sampling and handling soil and fruitbodies.

Compared to the effect of soil pH, the effect of fungal identity on the structure of bacterial communities in mushrooms was secondary but significant. In particular, we found that fungal taxonomy at the genus and order level significantly determines bacterial community structure in mushrooms. These results point to some level of habitat specificity and possibly specific functions of certain bacteria depending on the taxonomy and lifestyle of mushrooms. Indeed, a strong selection of specific bacterial communities has been observed in the ectomycorrhizosphere of truffles and other EcM fungi (Marupakula et al., 2015; Deveau et al., 2016). Similar to what has been observed in plants (Bulgarelli et al., 2013; Vandenkoornhuyse et al., 2015), variation of mushroom carbon-compounds or differential carbon allocation across different fungal groups (Boersma et al., 2009, 2010; Warmink et al., 2009) may mediate bacterial–fungus associations. For example, the difference in bacterial communities in mycorrhizae formed by *Suillus bovinus* and *Paxillus involutus* were ascribed to the preference of either mannitol or fructose, produced in the mycorrhizosphere of respective fungi (Timonen et al., 1998). Similarly, glycerol released by *Laccaria proxima* attracts *Variovorax paradoxus*-like bacteria in the mycosphere (Boersma et al., 2010). The initial selection of bacteria probably depends on soil, especially the mycosphere properties (Warmink et al., 2009), whereas the second step may be determined by fruitbody characteristics, such as the presence of different metabolites, compounds, pH (Danell et al., 1993), etc. In both fungal structures, bacteria exploit fungal exudates on hyphal surface, leaving fungal cells intact (Danell, 1994; Timonen et al., 1998). The exudation of fungal C-compounds, in turn, has been found to depend on environmental conditions, considerably decreasing at lower pH and temperature (Rangel-Castro and Danell, 2002), which may affect bacterial groups that depend on these compounds. Also in case of arbuscular mycorrhizal fungi, exudates from their spores have been suggested to explain the key role of the identity of these fungi in shaping the microbial community surrounding their spores (Iffis et al., 2016).

### Distinctness of Fungal-Associated Bacterial Communities

Our results add new evidence to support the notion of the common occurrence of selected bacterial taxa in different structures formed by fungi. Dominant bacteria detected here in mushrooms suggest that similarly to the ectomycorrhizosphere (Burke et al., 2008; Uroz et al., 2012; Deveau et al., 2016), EcM fungi tend to share a core bacterial community in their fruitbodies. Among the latter, based on HTS, the most evident common trend in bacterial communities of truffles (Antony-Babu et al., 2014; Benucci and Bonito, 2016) and mushrooms is the high abundance of Proteobacteria and Bacteroidetes, but the exclusion/paucity of Acidobacteria, Actinobacteria, and Planctomycetes, despite their abundance in the soils of all sampling localities. Low abundance of some of these taxa, as well as Flavobacteria, Firmicutes, or Verrucomicrobia in fruitbodies is shared with EcM formed by different fungi (Vik et al., 2013; Deveau et al., 2016). Differences between soil and fungal-associated bacterial communities may be related to lower nutritional status of soil compared to the so called hot spots created by fungal hyphae in soil (Nazir et al., 2010). It has been shown that the abundance of Proteobacteria is stimulated by higher nutritional status of soil in contrast to, e.g., *Acidobacteria* which prefer low-nutrient soils (Torsvik and Øvreås, 2002). Thus it is likely that the dominance of Proteobacteria in hyphae (Cho et al., 2003), fruitbodies (Barbieri et al., 2005; Dahm et al., 2005; Zagriadskaia et al., 2013), and mycorrhizal roots (Poole et al., 2001; Frey-Klett et al., 2007) is due to elevated carbon content in these fungal habitats.

Despite the dominance of Proteobacteria in fungus-associated bacterial communities, there seems to be substantial variation among its inclusive lower level and some other bacterial groups, relating to the identity, structure or lifestyle of the fungal host. For example, Alphaproteobacteria (mostly Bradyrhizobiaceae) dominated in fruitbodies of various *Tuber* species (Antony-Babu et al., 2014; Benucci and Bonito, 2016) and EcM of several fungi (Kataoka et al., 2008; Uroz et al., 2012; Deveau et al., 2016) but was not abundant in studied mushrooms, in *Tuber aestivum* EcM (Gryndler et al., 2013) or in other truffle genera (Benucci and Bonito, 2016). By contrast, *Pseudomonas* was one of the most common genera in mushrooms studied by us and earlier authors (Dahm et al., 2005) as well as in the casing layer of cultivated *Agaricus bisporus* (Zaranejad et al., 2012) but has been reported to be absent or very rare in truffles (Antony-Babu et al., 2014; Benucci and Bonito, 2016). Members of *Burkholderia*, frequently found in mycorrhizae (Timonen et al., 1998; Sbrana et al., 2002), appeared to be especially affiliated to *Lactarius*. Namely, these were by far the most abundant bacteria in fruitbodies of *L. rufus*, as evidenced here, but also common in EcM formed by *L. rufus* and *Pinus sylvestris* (Poole et al., 2001). *Rhizobium* and *Chitinophaga* fit the category of “specific fungiphiles” (Warmink et al., 2009) occurring almost exclusively in *Cantharellus*.

The effect of host identity on the structure of bacterial communities, revealed in this study, was largely (but not exclusively) driven by the distinct bacterial composition in fruitbodies of *C. cibarius.* The chanterelle belongs to the order *Cantharellales* that represents one of the early-diverged lineages of Agaricomycetes. It is thus phylogenetically most distant among mushroom-forming basidiomycetes, which is also reflected in the physiology and key compounds produced by members of this order (Rangel-Castro and Danell, 2002). Besides the high concentration of ergocalciferol (Rangel-Castro et al., 2002b) and accumulation of some metals from the soil (Drewnowska and Falandysz, 2015), fruitbodies of *C. cibarius* are known to produce antimicrobial compounds (Aina et al., 2012) that suppress the growth of some bacterial species (Barros et al., 2008). The distinct chemistry may explain the unique bacterial community of *C. cibarius*, which unlike other mushrooms, was dominated by members of the phylum Bacteroidetes (Supplementary Table S3). It is tempting to hypothesize that certain bacteria enriched in antibiotic resistance genes may be able to thrive in chanterelles.

### Methodological Considerations for Detection of Bacteria

Our results advocate the combined use of HTS and culturing in characterizing bacterial communities, in line with studies revealing differences in microbial diversity detected by the two methods in different environments (Vaz-Moreira et al., 2011; Stefani et al., 2015). While HTS by far surpasses culturing in revealing bacterial diversity, the latter can detect certain taxa missed by the former method and allow more precise identification of bacterial taxonomic and functional diversity. Consistent method-driven discrepancies can be discerned in revealing the structure of the bacterial communities of the studied epigeious basidiomycetes and hypogeous ascomycetes available for comparison, despite their different taxonomic identity and lifestyle. In particular, Gammaproteobacteria dominate among bacterial cultures from studied mushrooms as well as truffles but are much less frequently detected by culture-independent methods (Barbieri et al., 2005, 2007). By contrast, the latter revealed that Alphaproteobacteria occurred in mushrooms in this study, in truffles (Antony-Babu et al., 2014; Benucci and Bonito, 2016) and in truffle-like ascomycetes (Quandt et al., 2015), despite being absent or rarely isolated in culture. Similar to what has been reported for truffles, we detected *Actinobacteria* with similar frequency based on both methods, whereas members of *Firmicutes* were common in culture but rare in HTS data.

Evidence presented here suggests the potential of HTS in contrast with culture-based methods to capture differences among bacterial communities of different host taxa and the effect of underlying factors shaping their structure. The best example was provided by the bacterial community of the chanterelle that was clearly distinct from those of other mushrooms based on the dominance of Chitinophagaceae/Sphingobacteriales/Bacteroidetes in the HTS data. However, culturing revealed that these taxa were rare in all mushrooms, bacterial isolates of which, including *C. cibarius*, were dominated by Gammaproteobacteria.

## Conclusions

This study presents the first assessment of the structure of bacterial communities in mushrooms using HTS methods. Our findings support the hypothesis that bacterial communities in mushrooms are to a large extent affected by the same abiotic factors that shape bacterial communities in the surrounding soil, which is likely a major source of bacteria in mushrooms. Nevertheless, we found a significant and strong effect of fungal identity on the structure of their bacterial communities. This suggests that at least some bacteria may have specific symbiotic functions in different groups of mushrooms. In addition, conditions provided in fruitbodies may differ among fungal groups, resulting in habitat filtering of certain bacterial groups. Further studies are needed to explicitly test these hypotheses.

## Author Contributions

MP participated in study design, in collecting samples, in conducting molecular and statistical analyses, and in writing the manuscript. KP participated in study design, in collecting samples, in conducting molecular analyses, and in writing the manuscript. MB participated in study design, conducting statistical analyses, and in writing the manuscript.

## Conflict of Interest Statement

The authors declare that the research was conducted in the absence of any commercial or financial relationships that could be construed as a potential conflict of interest.

## Footnotes

1 http://mafft.cbrc.jp/alignment/server/index.html

2 https://www.arb-silva.de

3 http://greengenes.lbl.gov

4 http://rdp.cme.msu.edu/
