# Supplementary material for: Bacterial Communities in Boreal Forest Mushrooms Are Shaped Both by Soil Parameters and Host Identity
Source: Front Microbiol. 2017 May 10;8:836. doi: 10.3389/fmicb.2017.00836 (PMC5423949; doi:10.3389/fmicb.2017.00836)
Supplement: Supplementary file 1 [file Data_Sheet_1.pdf]

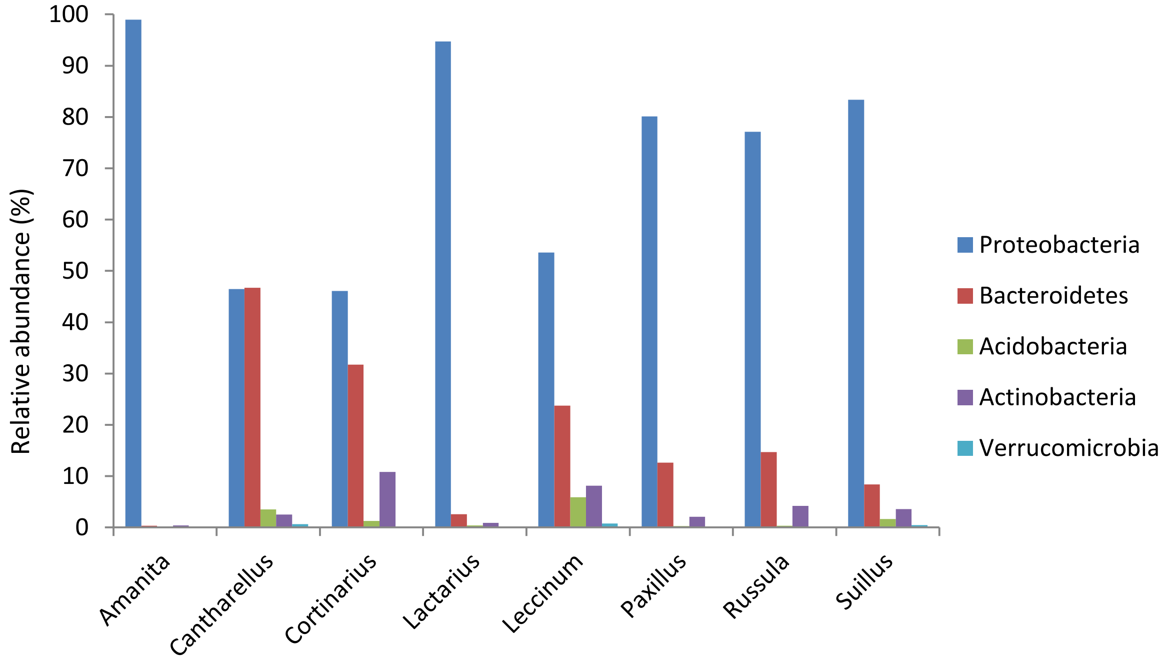

Figure S1. Relative abundance (%) of five most numerous bacterial phyla in eight mushroom genera based on HTS read numbers normalized by the total number of reads in each fungal genus.

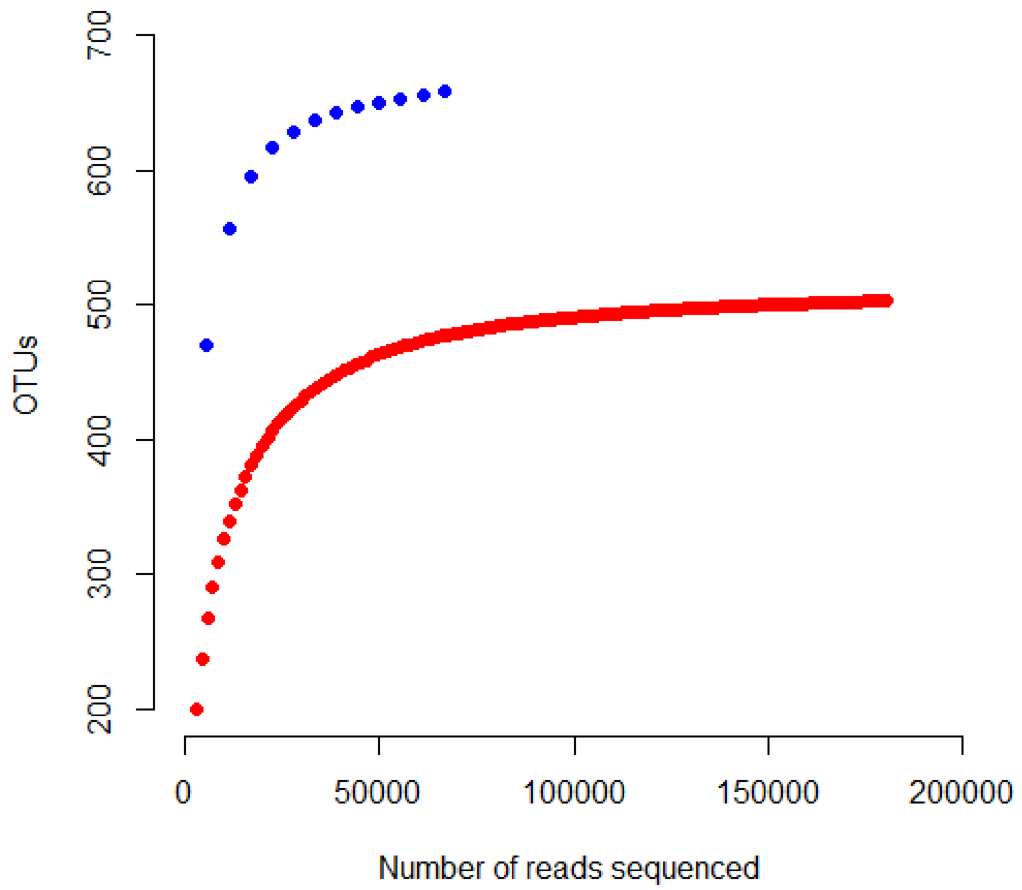

Figure S2. OTUs rarefication curves for the mushroom and soil samples based on HTS data.

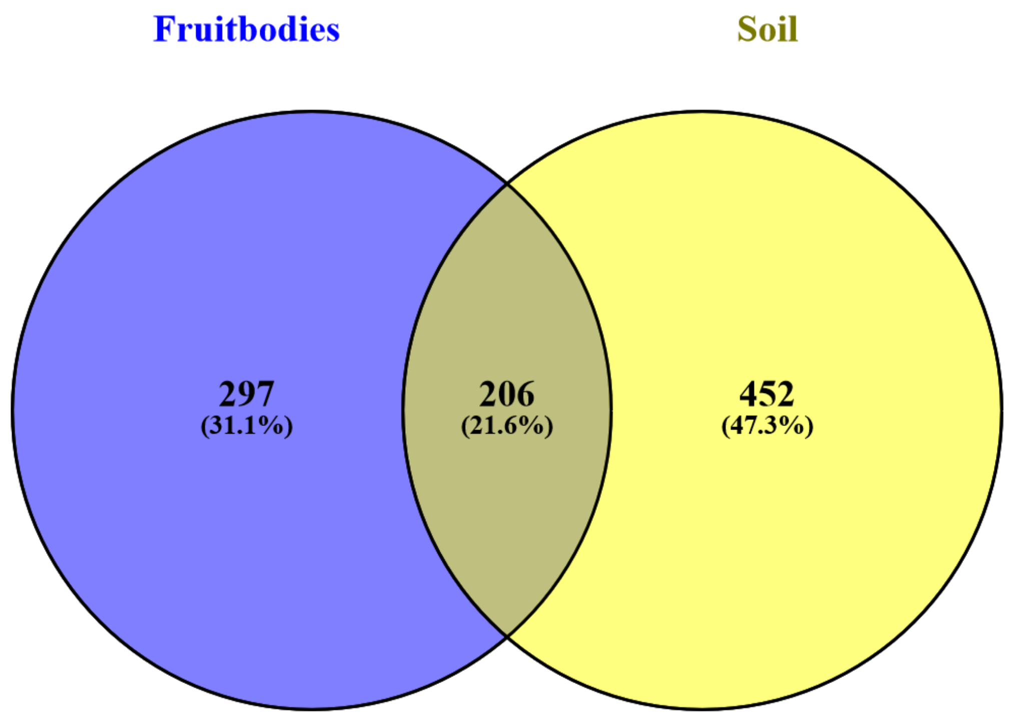

Figure S3. The number of OTUs found in fruitbodies, in soil and in both environments.

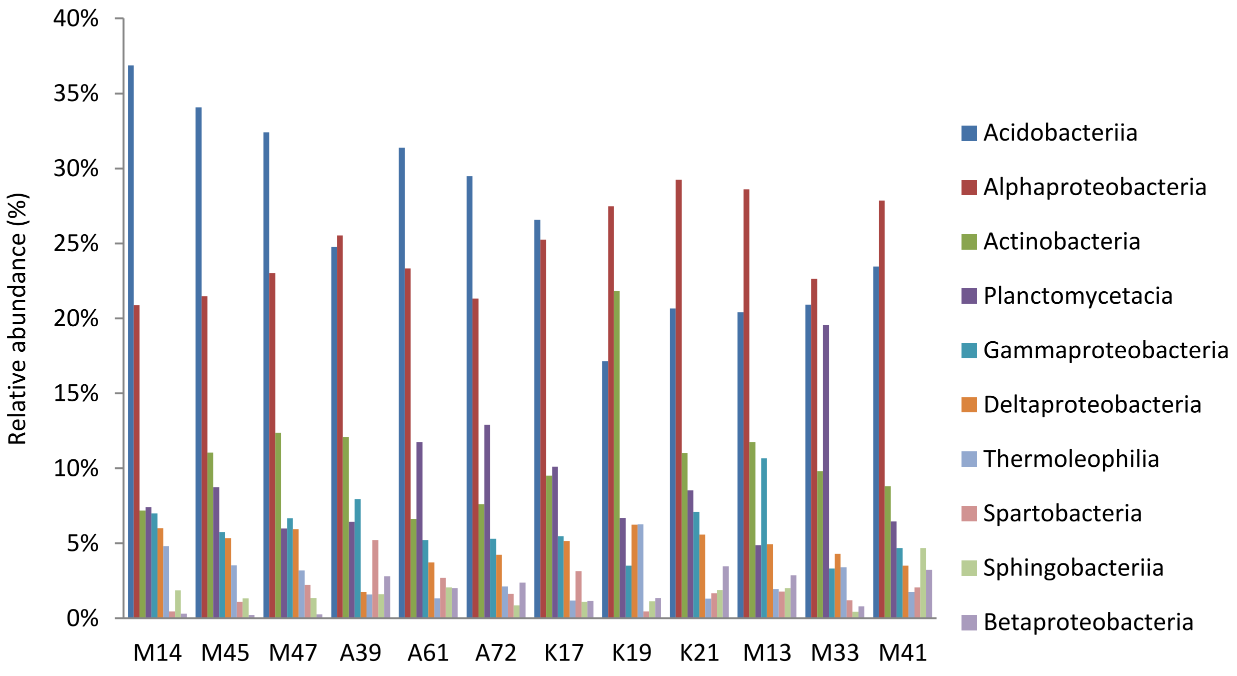

Figure S4. Relative abundance (%) of the ten most numerous bacterial classes at the 12 study sites based on HTS numbers normalized by the total number of reads from each site.

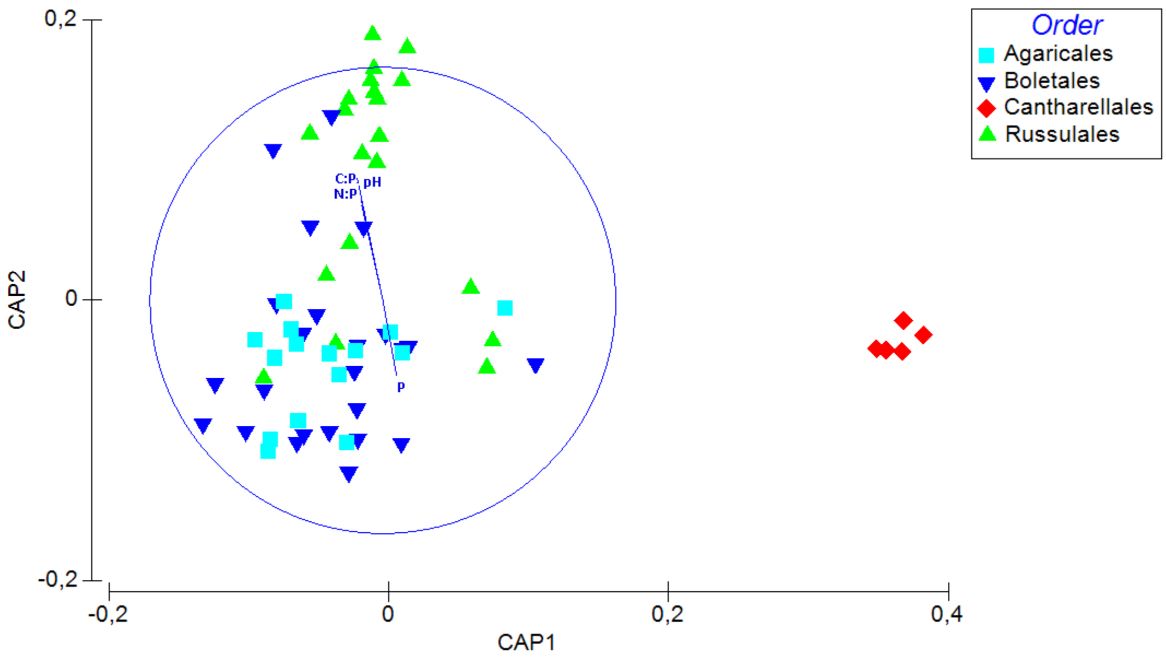

Figure S5. A plot of the canonical analysis of principal (CAP) coordinates visualising the differences in bacterial communities among fungal orders (analysed against the factor order) and the effect of soil parameters underlying the observed variation based on Illumina data. Vectors show Pearson correlations with soil variables (pH, C:P ratio, N:P ratio and P content) along the second axis of CAP (variables with correlations ≥ 0.3 are represented). Correlation coefficients for pH, P content, N:P and C:P ratio are 0.53, -0.33, 0.43 and 0.44, respectively.

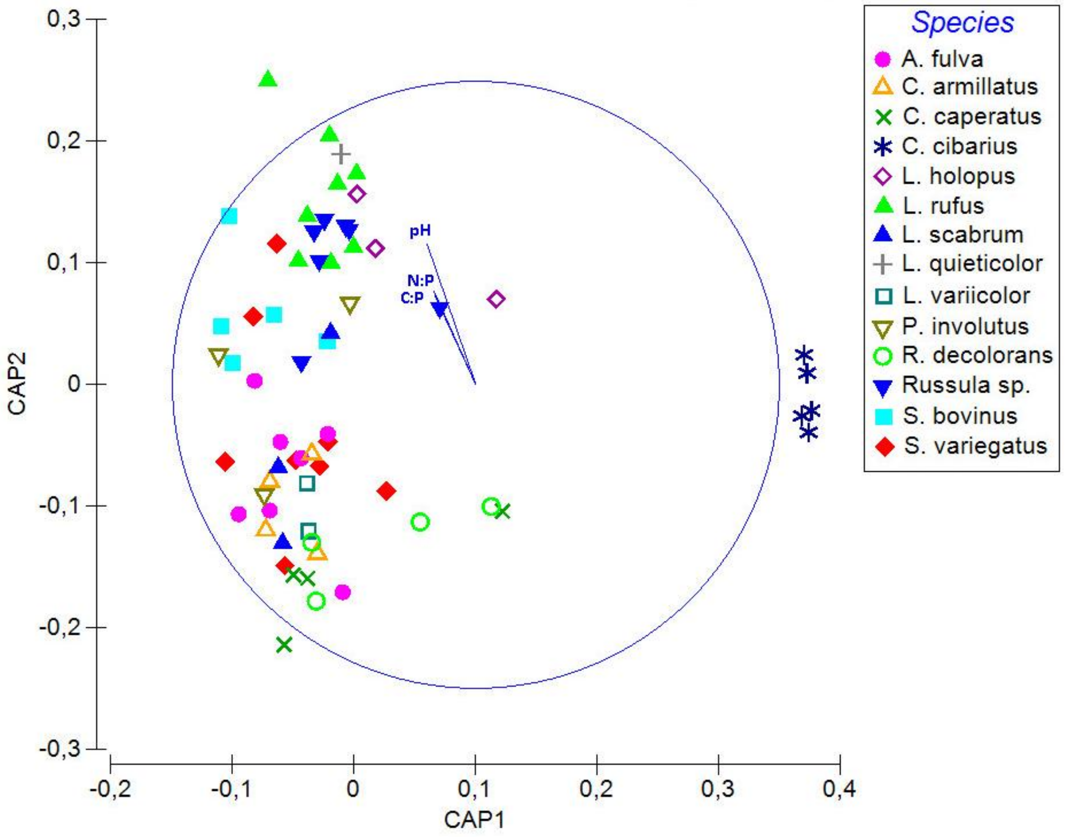

Figure S6. A plot of the canonical analysis of principal (CAP) coordinates visualising the differences in bacterial communities among fungal species (analysed against the factor species) and the effect of soil parameters underlying the observed variation based on Illumina data. Vectors show Pearson correlations with soil variables (pH, C:P ratio and N:P ratio) along the second axis of CAP (variables with correlations ≥ 0.3 are represented).

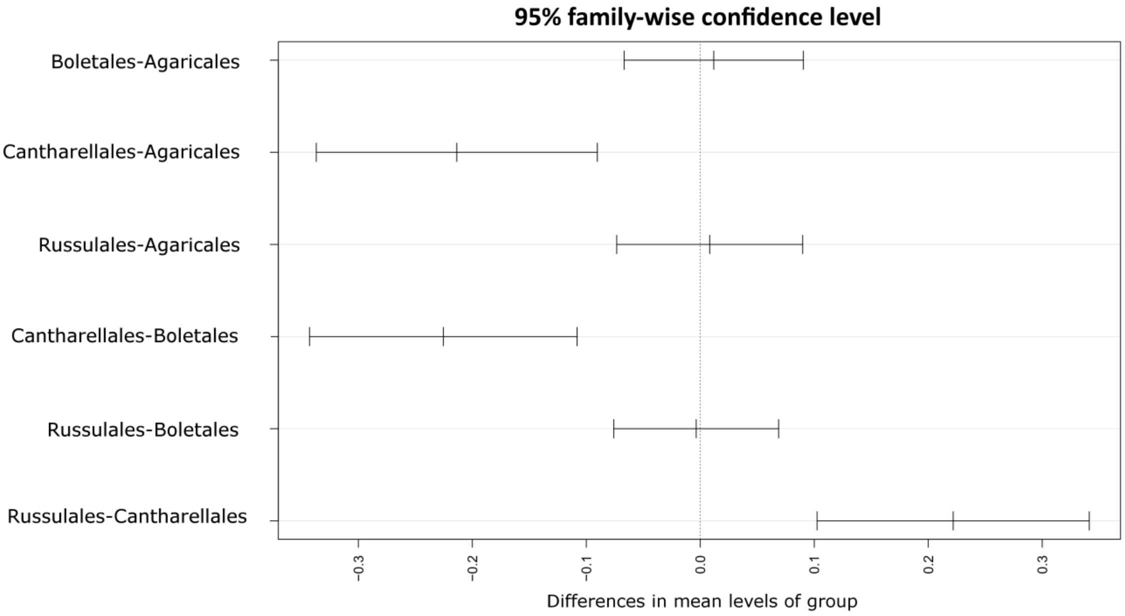

Figure S7. Results of Tukey's test showing statististically significant differences between studied fungal orders with respect to their bacterial community composition. Intervals that do not cross the line in the middle present a significant difference ($p<0.05$) between the bacterial communities of the two fungal orders.
