# Supplementary material for: Bacterial Communities in Boreal Forest Mushrooms Are Shaped Both by Soil Parameters and Host Identity
Source: Front Microbiol. 2017 May 10;8:836. doi: 10.3389/fmicb.2017.00836 (PMC5423949; doi:10.3389/fmicb.2017.00836)
Supplement: Supplementary file 2 [file Data_Sheet_2.pdf]

Table S1. Information on the 12 study sites and fungal species sampled at each. The numbers of fruitbodies analysed using high-throughput sequencing and culturing are shown in parantheses.

Nature reserve	Habitat type	Location	Site code	Year	Number of fruitbodies sampled from each mushroom species[a] at different sites<br>High-throughput sequencing	Culturing
**Meenikunno**	*Cladina*	57°56'17.2"N 27°24'12.6"E	M13	2014/ 2015	*C. armillatus* (2), *C. caperatus* (3), *Ca. cibarius* (1), *La. rufus* (4), *P. ivolutus* (3), *S. bovinus* (6), *S. variegatus* (3)	*A. muscaria* (1), *A. rubescens* (1), *C. armillatus* (2), *C. caperatus* (2), *Ca. cibarius* (1), *La. rufus* (8), *R. decolorans* (1), *Russula* spp. (2), *S. bovinus* (9), *S. variegatus* (3)
**Meenikunno**	*Cladina*	57°56'20.0"N 27°21'07.2"E	M47	2015	*A. fulva* (3), *C. armillatus* (2), *C. caperatus* (4), *Ca. cibarius* (4), *L. scabrum* (4), *R. decolorans* (3), *S. variegatus* (3)	*A. fulva* (3), *C. armillatus* (2), *C. caperatus* (2), *Ca. cibarius* (4), *La. rufus* (1), *L. scabrum* (2), *R. decolorans* (3)
**Karula**	*Cladina*	57°38'44.5"N 26°24'53.3"E	K17	2014	*La. rufus* (3), *Russula spp. (*3), *S. variegatus* (3)	*La. rufus* (3), *R. decolorans* (1), *Russula* spp. (1), *S. variegatus* (3)
**Agusalu**	*Cladina*	59°03'42.8"N 27°30'58.3"E	A39	2014	*La. rufus* (3), *Russula spp.* (3), *S. bovinus* (3)	*La. rufus* (3), *Russula* spp. (3)
**Meenikunno**	*Vaccinium myrtillus*	57°56'19.0"N 27°21'51.5"E	M41	2014/ 2015	*A. fulva* (3), *C. armillatus* (3), *Ca. cibarius* (2), *La. rufus* (3), *L. holopus* (2), *L. scabrum* (2), *P. involutus* (3), *R. decolorans* (3), *Russula spp. (*3), *S. variegatus* (2)	*A. fulva* (3), *C. armillatus* (2), *Ca. cibarius* (2), *La. rufus* (3), *L. holopus* (2), *L. scabrum* (1), *P. involutus* (2), *R. decolorans* (3), *Russula* spp. (1), *S. variegatus* (4)

**Meenikunno**	*Vaccinum myrtillus*	57°57'36.7"N 27°17'10.3"E	M45	2015	*C. caperatus* (4), *Ca. cibarius* (2), *R. decolorans* (2), *S. bovinus* (4), *S. variegatus* (1)	*Ca. cibarius* (2), *R. decolorans* (2), *S. bovinus* (2)
**Karula**	*Vaccinium myrtillus*	57°39'14.8"N 26°29'49.9"E	K21	2014	*A. fulva* (3), *La. rufus* (3), *Russula spp. (*3), *S. variegatus* (2)	*A. fulva* (3), *La. rufus* (3), *Russula* spp. (3), *S. variegatus* (2)
**Agusalu**	*Vaccinium myrtillus*	59°03'28.4"N 27°30'06.8"E	A61	2014	*La. rufus* (3), *Russula spp.* (3), *S. variegatus* (3)	*La. rufus* (2), *R. decolorans* (1), *Russula* spp. (1)
**Meenikunno**	*Polytrichum*	57°57'01.4"N 27°22'07.7"E	M33	2014/ 2015	*A. fulva* (1), *C. armillatus* (3), *C. caperatus* (3), *Ca. cibarius* (3), *La. rufus* (3), *L. holopus* (3), *L. scabrum* (2), *L. variicolor* (4), *P. involutus* (3), *S. bovinus* (3)	*A. fulva* (8), *C. armillatus* (3), *C. caperatus* (2), *Ca. cibarius* (3), *La. rufus* (12), *L. holopus* (3), *L. scabrum* (1), *L. variicolor* (1), *P. involutus* (3), *Russula* spp. (3), *S. bovinus* (3), *S. variegatus* (3)
**Meenikunno**	*Polytrichum*	57°55'16.3"N 27°22'44.8"E	M14	2015	*A. fulva* (2), *L. holopus* (2), *L. variicolor* (2), *R. decolorans* (3)	*L. holopus* (2), *L. variicolor* (2), *R. decolorans* (2)
**Karula**	*Polytrichum*	57°38'29.4"N 26°24'53.6"E	K19	2014	*A. fulva* (3), *La. rufus* (3), *Russula spp.* (3), *S. bovinus* (1), *S. variegatus* (2)	*A. fulva* (3), *La. rufus* (3), *Russula* spp. (2), *S. bovinus* (1), *S. variegatus* (2)
**Agusalu**	*Polytrichum*	59°03'19.1"N 27°30'26.6"E	A72	2014	*A. fulva* (3), *La. quieticolor* (3), *Russula spp.* (3), *S. variegatus* (3)	*A. fulva* (3), *La. quieticolor* (3), *Russula* spp. (3), *S. variegatus* (1)

[a]Names of fungal genera are abbreviated as follows: *A – Amanita, Ca – Cantharellus, C – Cortinarius, La – Lactarius, L – Leccinum, P – Paxillus, R – Russula, S – Suillus.*

Table S2. Soil parameters and soil type info for the 12 studied sites used in statistical analysis.

Site	Organic matter* (%)	N* (%)	C* (%)	C:N Observed/ measured	P * (mg/kg)	K * (mg/kg)	Ca* (mg/kg)	Mg* (mg/kg)	δ 15N	N:P	C:P	C:N calculat ed	pH_{KCl}	Soil Type[1]
**M13**	4.74	0.07	2.71	38.12	8.26	26.66	68.18	15.42	-0.44	0.01	0.33	37.92	2.86	LIII
**M33**	59.45	0.8	24.24	30.16	92.57	361.95	375.69	185.68	-1.2	0.01	0.26	30.16	2.80	LG1
**M41**	11.38	0.19	6.03	31.1	6.81	121.29	179.95	41.98	-0.14	0.02	0.89	31.12	2.46	L(k)II
**M45**	19.04	0.38	11.71	30.78	13.91	109.32	128.25	34.58	0.1	0.03	0.84	30.78	2.62	LG
**M47**	7.29	0.40	13.88	34.73	26.33	48.29	125.21	20.85	-1.80	0.02	0.53	34.73	2.63	LI
**M14**	24.10	0.53	13.65	25.89	19.85	128.48	641.96	162.67	0.54	0.03	0.69	25.89	2.91	LG
**K17**	2.57	0.15	4.91	34	2.06	51.24	76.91	10.72	-1.07	0.07	2.39	33.77	2.84	LII
**K19**	18.34	0.85	24.96	29.52	16.3	134.67	183.86	47.3	1.05	0.05	1.53	29.52	3.07	LG1

**K21**	12.59	0.13	4.23	31.61	13.35	77.24	129.62	39.97	0.28	0.01	0.32	31.61	3.08	LG
**A39**	5.58	0.08	2.75	34.84	1.11	44.25	126.99	15.68	-1.59	0.07	2.48	34.69	3.47	LI
**A61**	12.48	0.28	9.99	35.24	10.89	176.61	230.75	38.61	1.89	0.03	0.92	35.46	3.36	LG
**A72**	20.89	0.3	9.39	31.1	1.01	171.99	221.66	48.91	1.01	0.02	0.64	31.30	2.94	LG1

*Log-transformed for statistical analyses

[1] LI-podzols, no humus layer, albic horizon < 7 cm, no gleyic features; LII, L(k)II-podzols, albic horizon > 7 cm, no gleyic features; LG-gleysols, moderately gleyic features, peat layer < 10 cm; LG1-gleysols, strongly gleyic features, peat layer 10-30 cm
