# Supplementary material for: Bacterial Communities in Boreal Forest Mushrooms Are Shaped Both by Soil Parameters and Host Identity
Source: Front Microbiol. 2017 May 10;8:836. doi: 10.3389/fmicb.2017.00836 (PMC5423949; doi:10.3389/fmicb.2017.00836)
Supplement: Supplementary file 3 [file Data_Sheet_3.pdf]

Table S3. Taxonomic classification of bacterial isolates based on identification of the most similar 16S rDNA sequences at the international nucleotide sequence databases; species names are missing if no >97% similar sequences were found.

**Sample Number/ GenBank accession number**	**Fruitbody code**	**MUSHROOMS**		**Site**	**BACTERIA**					
		Genus	Species		Phylum	Class	Order	Family	Genus	Species
**136./ KY682059**	R117	Russula	paludosa	K17	Actinobacteria	Actinobacteria	Micrococcales	Microbacteriaceae	Frondicola	Frondicola australicus (97.91%)
**274_1492R*/ KY682057**	C.c.1/M33	Cantharellus	cibarius	M33	Actinobacteria	Actinobacteria	Micrococcales	Microbacteriaceae	unclassif.Microbacteriaceae	unclassif.Microbacteriaceae
**113./ KY682058**	R121	Russula	rhodopus	K21	Actinobacteria	Actinobacteridae	Micrococcales	Microbacteriaceae	Microbacterium	Microbacterium aurum (98.05%)
**58.*/ KY681818**	A321	Amanita	fulva	K21	Bacteroidetes	Flavobacteriia	Flavobacteriales	Flavobacteriaceae	Flavobacterium (96.03%)	unclassif.Flavobacterium sp.
**18./ KY681819**	A333	Amanita	fulva	M33	Bacteroidetes	Sphingobacteriia	Sphingobacteriales	Sphingobacteriaceae	Sphingobacterium	Sphingobacterium faecium (97.49%)
**263. 1492R / KY681820**	C.c.1/M41	Cantharellus	cibarius	M41	Bacteroidetes	Sphingobacteriia	Sphingobacteriales	Sphingobacteriaceae	Pedobacter (96%)	unclassif.Pedobacter sp.
**98./ KY682069**	R339	Russula	emetica	A39	Firmicutes	Bacilli	Bacillales	Staphylococcaceae	Staphylococcus	Staphylococcus epidermidis (100.00%)
**107./ KY682065**	S841	Suillus	variegatus	M41	Firmicutes	Bacilli	Bacillales	Staphylococcaceae	Staphylococcus	Staphylococcus pasteuri (99.93%)
**111./ KY682066**	R317	Russula	decolorans	K17	Firmicutes	Bacilli	Bacillales	Staphylococcaceae	Staphylococcus	Staphylococcus pasteuri (100.00%)
**112./ KY682067**	R221	Russula	vinosa	K21	Firmicutes	Bacilli	Bacillales	Staphylococcaceae	Staphylococcus	Staphylococcus pasteuri (99.86%)
**138./ KY682068**	M13-C5	Cortinarius	caperatus	M13	Firmicutes	Bacilli	Bacillales	Staphylococcaceae	Staphylococcus	Staphylococcus epidermidis (100.00%)
**222. 1492R/ KY682064**	P.invol.3/M41	Paxillus	involutus	M41	Firmicutes	Bacilli	Bacillales	Staphylococcaceae	Staphylococcus	Staphylococcus pasteuri (99.145)
**94./ KY682061**	L272	Lactarius	quieticolor	A72	Proteobacteria	Alphaproteobacteria	Rhizobiales	Rhizobiaceae	Rhizobium	Rhizobium rhizogenes (99.85%)
**C_124. 1492R/ KY682060**	R239	Russula	emetica	A39	Proteobacteria	Alphaproteobacteria	Rhizobiales	Rhizobiaceae	Rhizobium	Rhizobium rhizogenes (99.89%)
**130./ KY682062**	R172	Russula	emetica	A72	Proteobacteria	Alphaproteobacteria	Rhizobiales	Brucellaceae	Pseudochrobactrum	Pseudochrobactrum kiredjianiae (98.14%)
**120./ KY682063**	R941	Russula	decolorans	M41	Proteobacteria	Alphaproteobacteria	Sphingomonadales	Sphingomonadaceae	Sphingomonas	Sphingomonas dokdonensis (100.00%)
**34./ KY681959**	S117	Suillus	variegatus	K17	Proteobacteria	Betaproteobacteria	Burkholderiales	Burkholderiaceae	Burkholderia	Burkholderia sordidicola (99.93%)
**45./ KY681969**	S117	Suillus	variegatus	K17	Proteobacteria	Betaproteobacteria	Burkholderiales	Burkholderiaceae	Burkholderia	Burkholderia phenazinium (99.35%)
**47./ KY681971**	L261	Lactarius	rufus	A61	Proteobacteria	Betaproteobacteria	Burkholderiales	Burkholderiaceae	Burkholderia	Burkholderia xenovorans

										(98.71%)
**48./ KY681990**	L372	Lactarius	quieticolor	A72	Proteobacteria	Betaproteobacteria	Burkholderiales	Burkholderiaceae	Burkholderia	Burkholderia phenazinium (98.92%)
**54./ KY681993**	L239	Lactarius	rufus	A39	Proteobacteria	Betaproteobacteria	Burkholderiales	Burkholderiaceae	Burkholderia	Burkholderia phytofirmans (98.28%)
**55./ KY681995**	L221	Lactarius	rufus	K21	Proteobacteria	Betaproteobacteria	Burkholderiales	Burkholderiaceae	Burkholderia	Burkholderia phytofirmans (98.28%)
**61./ KY681970**	S213	Suillus	variegatus	M13	Proteobacteria	Betaproteobacteria	Burkholderiales	Burkholderiaceae	Burkholderia	Burkholderia sordidicola (99.57%)
**67./ KY681997**	L217	Lactarius	rufus	K17	Proteobacteria	Betaproteobacteria	Burkholderiales	Burkholderiaceae	Burkholderia	Burkholderia phytofirmans (98.28%)
**79./ KY682025**	L139	Lactarius	rufus	A39	Proteobacteria	Betaproteobacteria	Burkholderiales	Burkholderiaceae	Burkholderia	Burkholderia phenazinium (98.63%)
**80./ KY682000**	L121	Lactarius	rufus	K21	Proteobacteria	Betaproteobacteria	Burkholderiales	Burkholderiaceae	Burkholderia	Burkholderia phytofirmans (98.28%)
**81./ KY681998**	S533	Suillus	variegatus	M33	Proteobacteria	Betaproteobacteria	Burkholderiales	Burkholderiaceae	Burkholderia	Burkholderia phytofirmans (98.28%)
**82./ KY682028**	S433	Suillus	variegatus	M33	Proteobacteria	Betaproteobacteria	Burkholderiales	Burkholderiaceae	Burkholderia	Burkholderia megapolitana(98.49%)
**83./ KY682029**	S313	Suillus	variegatus	M13	Proteobacteria	Betaproteobacteria	Burkholderiales	Burkholderiaceae	Burkholderia	Burkholderia phenazinium (99.57%)
**85./ KY682024**	S113	Suillus	variegatus	M13	Proteobacteria	Betaproteobacteria	Burkholderiales	Burkholderiaceae	Burkholderia	Burkholderia bryophila (98.35%)
**86./ KY681999**	L321	Lactarius	rufus	K21	Proteobacteria	Betaproteobacteria	Burkholderiales	Burkholderiaceae	Burkholderia	Burkholderia phytofirmans (98.28%)
**87./ KY681994**	L339	Lactarius	rufus	A39	Proteobacteria	Betaproteobacteria	Burkholderiales	Burkholderiaceae	Burkholderia	Burkholderia phytofirmans (98.28%)
**88./ KY681996**	L319	Lactarius	rufus	K19	Proteobacteria	Betaproteobacteria	Burkholderiales	Burkholderiaceae	Burkholderia	Burkholderia phytofirmans (98.28%)
**89./ KY682037**	L317	Lactarius	rufus	K17	Proteobacteria	Betaproteobacteria	Burkholderiales	Burkholderiaceae	Burkholderia	Burkholderia caledonica (98.57%)
**92./ KY681960**	L117	Lactarius	rufus	K17	Proteobacteria	Betaproteobacteria	Burkholderiales	Burkholderiaceae	Burkholderia	Burkholderia sordidicola (99%)
**95./ KY682043**	L172	Lactarius	quieticolor	A72	Proteobacteria	Betaproteobacteria	Burkholderiales	Oxalobacteraceae	Collimonas	Collimonas fungivorans (99.57%)
**96./ KY682046**	L172	Lactarius	quieticolor	A72	Proteobacteria	Betaproteobacteria	Burkholderiales	Comamonadaceae	Variovorax	Variovorax paradoxus (99.86%)
**114./ KY682042**	R272	Russula	emetica	A72	Proteobacteria	Betaproteobacteria	Burkholderiales	Burkholderiaceae	Pandoraea	Pandoraea norimbergensis (99.64%)
**122./ KY681985**	R533	Russula	emetica	M33	Proteobacteria	Betaproteobacteria	Burkholderiales	Burkholderiaceae	Burkholderia	Burkholderia bryophila (100.00%)
**135./ KY682022**	R633	Russula	emetica	M33	Proteobacteria	Betaproteobacteria	Burkholderiales	Burkholderiaceae	Burkholderia	Burkholderia xenovorans (98%)
**137./ KY682030**	S533	Suillus	variegatus	M33	Proteobacteria	Betaproteobacteria	Burkholderiales	Burkholderiaceae	Burkholderia	Burkholderia phenazinium (99.50%)
**140./ KY682003**	M13-4	Lactarius	rufus	M13	Proteobacteria	Betaproteobacteria	Burkholderiales	Burkholderiaceae	Burkholderia	Burkholderia phytofirmans (98.28%)
**141./ KY681987**	M13-6	Lactarius	rufus	M13	Proteobacteria	Betaproteobacteria	Burkholderiales	Burkholderiaceae	Burkholderia	Burkholderia bryophila

										(99.57%)
**142./KY682008**	M13-2	Lactarius	rufus	M13	Proteobacteria	Betaproteobacteria	Burkholderiales	Burkholderiaceae	Burkholderia	Burkholderia phytofirmans (98.28%)
**143./KY681972**	M33-6	Lactarius	rufus	M33	Proteobacteria	Betaproteobacteria	Burkholderiales	Burkholderiaceae	Burkholderia	Burkholderia cepacia (98.57%)
**144./KY681973**	M33-7	Lactarius	rufus	M33	Proteobacteria	Betaproteobacteria	Burkholderiales	Burkholderiaceae	Burkholderia	Burkholderia cepacia (98.78%)
**145./KY682001**	M33-5	Lactarius	rufus	M33	Proteobacteria	Betaproteobacteria	Burkholderiales	Burkholderiaceae	Burkholderia	Burkholderia phytofirmans (98.28%)
**147./KY681991**	L219	Lactarius	rufus	K19	Proteobacteria	Betaproteobacteria	Burkholderiales	Burkholderiaceae	Burkholderia	Burkholderia fungorum (99.35%)
**154./KY682039**	S633	Suillus	variegatus	M33	Proteobacteria	Betaproteobacteria	Burkholderiales	Burkholderiaceae	Burkholderia	Burkholderia xenovorans (98%)
**155./KY681988**	S941	Suillus	variegatus	M41	Proteobacteria	Betaproteobacteria	Burkholderiales	Burkholderiaceae	Burkholderia	Burkholderia bryophila (99.57%)
**156./KY682006**	R433	Russula	emetica	M33	Proteobacteria	Betaproteobacteria	Burkholderiales	Burkholderiaceae	Burkholderia	Burkholderia phytofirmans (98.28%)
**C. 158_1492R/ KY681958**	L933	Lactarius	rufus	M33	Proteobacteria	Betaproteobacteria	Burkholderiales	Burkholderiaceae	Burkholderia	Burkholderia xenovorans (98.91%)
**159./KY681975**	L833	Lactarius	rufus	M33	Proteobacteria	Betaproteobacteria	Burkholderiales	Burkholderiaceae	Burkholderia	Burkholderia cepacia (98.71%)
**161./KY681976**	L733	Lactarius	rufus	M33	Proteobacteria	Betaproteobacteria	Burkholderiales	Burkholderiaceae	Burkholderia	Burkholderia cepacia (98.92%)
**162./KY682040**	R113	Russula	paludosa	M13	Proteobacteria	Betaproteobacteria	Burkholderiales	Burkholderiaceae	Burkholderia	Burkholderia megapolitana (98.28%)
**163./KY682007**	L313	Lactarius	rufus	M13	Proteobacteria	Betaproteobacteria	Burkholderiales	Burkholderiaceae	Burkholderia	Burkholderia phytofirmans (98.28%)
**C_164. 1492R*/ KY681957**	L213	Lactarius	rufus	M13	Proteobacteria	Betaproteobacteria	Burkholderiales	Burkholderiaceae	Burkholderia	Burkholderia xenovorans (98.57%)
**166./KY681974**	M33-4	Lactarius	rufus	M33	Proteobacteria	Betaproteobacteria	Burkholderiales	Burkholderiaceae	Burkholderia	Burkholderia cepacia (98.85%)
**167./KY681961**	A239	Amanita	rubescens	A39	Proteobacteria	Betaproteobacteria	Burkholderiales	Burkholderiaceae	Burkholderia	Burkholderia sordidicola (98.92%)
**168. 1492R/ KY681989**	L. scabr.2/M47	Leccinum	scabrum	M47	Proteobacteria	Betaproteobacteria	Burkholderiales	Burkholderiaceae	Burkholderia	Burkholderia bryophila (98.60%)
**169. 1492R/ KY682019**	C.a.1/M41	Cortinarius	armillatus	M41	Proteobacteria	Betaproteobacteria	Burkholderiales	Burkholderiaceae	Burkholderia	Burkholderia graminis (98.25%)
**169./KY682004**	L1133	Lactarius	rufus	M33	Proteobacteria	Betaproteobacteria	Burkholderiales	Burkholderiaceae	Burkholderia	Burkholderia phytofirmans (98.28%)
**170. 1492R/ KY681983**	S.bov.15/M33	Suillus	bovinus	M33	Proteobacteria	Betaproteobacteria	Burkholderiales	Burkholderiaceae	Burkholderia	Burkholderia bryophila (99.44%)
**170./KY682002**	M13-7	Lactarius	rufus	M13	Proteobacteria	Betaproteobacteria	Burkholderiales	Burkholderiaceae	Burkholderia	Burkholderia phytofirmans (98.28%)
**171./KY682005**	M13-5	Lactarius	rufus	M13	Proteobacteria	Betaproteobacteria	Burkholderiales	Burkholderiaceae	Burkholderia	Burkholderia phytofirmans (98.28%)
**172./KY681986**	R372	Russula	emetica	A72	Proteobacteria	Betaproteobacteria	Burkholderiales	Burkholderiaceae	Burkholderia	Burkholderia bryophila (99.78%)
**174. 1492R/ KY681962**	L.variic.1/M14	Leccinum	variicolor	M14	Proteobacteria	Betaproteobacteria	Burkholderiales	Burkholderiaceae	Burkholderia	Burkholderia glathei (99.47%)

**175. 1492R/ KY681981**	L.variic.2/M14	Leccinum	variicolor	M14	Proteobacteria	Betaproteobacteria	Burkholderiales	Burkholderiaceae	Burkholderia	Burkholderia bryophila (99.63%)
**178. 1492R/ KY681964**	C.a.3/M33	Cortinarius	armillatus	M33	Proteobacteria	Betaproteobacteria	Burkholderiales	Burkholderiaceae	Burkholderia	Burkholderia glathei (99.56%)
**179. 1492R/ KY682027**	L.scabr.2/M41	Leccinum	scabrum	M41	Proteobacteria	Betaproteobacteria	Burkholderiales	Burkholderiaceae	Burkholderia	Burkholderia phenazinium (98.16%)
**184. 1492R/ KY682014**	L.scabr.3/M47	Leccinum	scabrum	M47	Proteobacteria	Betaproteobacteria	Burkholderiales	Burkholderiaceae	Burkholderia	Burkholderia graminis (98.42%)
**188. 1492R/ KY682035**	R.dec.1/M45	Russula	decolorans	M45	Proteobacteria	Betaproteobacteria	Burkholderiales	Burkholderiaceae	Burkholderia	Burkholderia phenazinium (98.16%)
**194. 1492R/ KY681980**	R.dec.60/M14	Russula	decolorans	M14	Proteobacteria	Betaproteobacteria	Burkholderiales	Burkholderiaceae	Burkholderia	Burkholderia bryophila (99.81%)
**197. 1492R/ KY682044**	R.dec.62/M14	Russula	decolorans	M14	Proteobacteria	Betaproteobacteria	Burkholderiales	Oxalobacteraceae	Janthinobacterium	Janthinobacterium agaricidamnosum (97%)
**198. 1492R/ KY682026**	R.dec.17/M47	Russula	decolorans	M47	Proteobacteria	Betaproteobacteria	Burkholderiales	Burkholderiaceae	Burkholderia	Burkholderia phenazinium (98.25%)
**200. 1492R/ KY682034**	R.dec.16/M47	Russula	decolorans	M47	Proteobacteria	Betaproteobacteria	Burkholderiales	Burkholderiaceae	Burkholderia	Burkholderia phenazinium (98.60%)
**205. 1492R/ KY682032**	S.bov.17/M33	Suillus	bovinus	M33	Proteobacteria	Betaproteobacteria	Burkholderiales	Burkholderiaceae	Burkholderia	Burkholderia phenazinium (99.39%)
**208. 1492R/ KY681977**	S.bov.16/M13	Suillus	bovinus	M13	Proteobacteria	Betaproteobacteria	Burkholderiales	Burkholderiaceae	Burkholderia	Burkholderia xenovorans (99%)
**209. 1492R/ KY682033**	S.bov.4/M45	Suillus	bovinus	M45	Proteobacteria	Betaproteobacteria	Burkholderiales	Burkholderiaceae	Burkholderia	Burkholderia phenazinium (98.51%)
**210. 1492R/ KY681968**	S.bov.15/M13	Suillus	bovinus	M13	Proteobacteria	Betaproteobacteria	Burkholderiales	Burkholderiaceae	Burkholderia	Burkholderia glathei (99.03%)
**214. 1492R/ KY682031**	Cort. cap.2/M47	Cortinarius	caperatus	M47	Proteobacteria	Betaproteobacteria	Burkholderiales	Burkholderiaceae	Burkholderia	Burkholderia phenazinium (98.51%)
**221. 1492R/ KY681965**	P.invol.3/M41	Paxillus	involutus	M41	Proteobacteria	Betaproteobacteria	Burkholderiales	Burkholderiaceae	Burkholderia	Burkholderia glathei (99.82%)
**227. 1492R/ KY682018**	P.invol.2/M33	Paxillus	involutus	M33	Proteobacteria	Betaproteobacteria	Burkholderiales	Burkholderiaceae	Burkholderia	Burkholderia graminis (98.33%)
**228. 1492R/ KY682041**	P.invol.2/M33	Paxillus	involutus	M33	Proteobacteria	Betaproteobacteria	Burkholderiales	Burkholderiaceae	Burkholderia	unclassif.Burkholderia sp.
**229. 1492R/ KY681992**	Cort.cap.75/M13	Cortinarius	caperatus	M13	Proteobacteria	Betaproteobacteria	Burkholderiales	Burkholderiaceae	Burkholderia	Burkholderia phytofirmans (98.51%)
**232. 1492R/ KY681966**	R.dec.52/M41	Russula	decolorans	M41	Proteobacteria	Betaproteobacteria	Burkholderiales	Burkholderiaceae	Burkholderia	Burkholderia glathei (99.82%)
**240. 1492R/ KY682010**	C.a.1/M33	Cortinarius	armillatus	M33	Proteobacteria	Betaproteobacteria	Burkholderiales	Burkholderiaceae	Burkholderia	Burkholderia xenovorans (97.90%)
**245. 1492R/ KY681984**	C.a.2/M47	Cortinarius	armillatus	M47	Proteobacteria	Betaproteobacteria	Burkholderiales	Burkholderiaceae	Burkholderia	Burkholderia bryophila (99.80%)
**247. 1492R/ KY681967**	C.a.2/M33	Cortinarius	armillatus	M33	Proteobacteria	Betaproteobacteria	Burkholderiales	Burkholderiaceae	Burkholderia	Burkholderia sordidcola (99.83%)

**253. 1492R/ KY682045**	C.c.2/M45	Cantharellus	cibarius	M45	Proteobacteria	Betaproteobacteria	Burkholderiales	Oxalobacteraceae	Herbaspirillum	Herbaspirillum rhizosphaerae (98.89%)
**267. 1492R/ KY682021**	C.a.2/M13	Cortinarius	armillatus	M13	Proteobacteria	Betaproteobacteria	Burkholderiales	Burkholderiaceae	Burkholderia	Burkholderia graminis (97.74%)
**268. 1492R/ KY682009**	C.a.1/M13	Cortinarius	armillatus	M13	Proteobacteria	Betaproteobacteria	Burkholderiales	Burkholderiaceae	Burkholderia	Burkholderia xenovorans (97.97%)
**271. 1492R/ KY682013**	P.invol.1/M33	Paxillus	involutus	M33	Proteobacteria	Betaproteobacteria	Burkholderiales	Burkholderiaceae	Burkholderia	Burkholderia graminis (98.42%)
**273. 1492R/ KY682012**	P.invol.2/M33	Paxillus	involutus	M33	Proteobacteria	Betaproteobacteria	Burkholderiales	Burkholderiaceae	Burkholderia	Burkholderia graminis (98.42%)
**281. 1492R/ KY681963**	C.a.2/M47	Cortinarius	armillatus	M47	Proteobacteria	Betaproteobacteria	Burkholderiales	Burkholderiaceae	Burkholderia	Burkholderia glathei (99.56%)
**286. 1492R/ KY681978**	C.a.2/M33	Cortinarius	armillatus	M33	Proteobacteria	Betaproteobacteria	Burkholderiales	Burkholderiaceae	Burkholderia	Burkholderia xenovorans (99.04%)
**287. 1492R/ KY682047**	C.c.1/M45	Cantharellus	cibarius	M45	Proteobacteria	Betaproteobacteria	Burkholderiales	Comamonadaceae	Variovorax	Variovorax paradoxus (99.86%)
**293. 1492R/ KY682038**	C.c.2/M45	Cantharellus	cibarius	M45	Proteobacteria	Betaproteobacteria	Burkholderiales	Burkholderiaceae	Burkholderia	Burkholderia bryophila (98.24%)
**295. 1492R/ KY682017**	L. holop.2/M14	Leccinum	holopus	M14	Proteobacteria	Betaproteobacteria	Burkholderiales	Burkholderiaceae	Burkholderia	Burkholderia graminis (98.25%)
**296. 1492R/ KY681979**	L.holop.2/M14	Leccinum	holopus	M14	Proteobacteria	Betaproteobacteria	Burkholderiales	Burkholderiaceae	Burkholderia	Burkholderia xenovorans (98.77%)
**304. 1492R/ KY682016**	L. holop.22/M33	Leccinum	holopus	M33	Proteobacteria	Betaproteobacteria	Burkholderiales	Burkholderiaceae	Burkholderia	Burkholderia phytofirmans (99%)
**309. 1492R/ KY681982**	L. scabr.17/M33	Leccinum	scabrum	M33	Proteobacteria	Betaproteobacteria	Burkholderiales	Burkholderiaceae	Burkholderia	Burkholderia bryophila (99.44%)
**313. 1492R/ KY682020**	S.v.15/M41	Suillus	variegatus	M41	Proteobacteria	Betaproteobacteria	Burkholderiales	Burkholderiaceae	Burkholderia	Burkholderia graminis (97.98%)
**315. 1492R/ KY682011**	L. scabr.3/M47	Leccinum	scabrum	M47	Proteobacteria	Betaproteobacteria	Burkholderiales	Burkholderiaceae	Burkholderia	Burkholderia xenovorans (97.81%)
**316. 1492R/ KY682036**	C.a.3/M33	Cortinarius	armillatus	M33	Proteobacteria	Betaproteobacteria	Burkholderiales	Burkholderiaceae	Burkholderia	Burkholderia phenazinium (99.04%)
**322. 1492R/ KY682015**	C.a.2/M13	Cortinarius	armillatus	M13	Proteobacteria	Betaproteobacteria	Burkholderiales	Burkholderiaceae	Burkholderia	Burkholderia graminis (98.33%)
**325. 1492R/ KY682023**	S.v.15/M41	Suillus	variegatus	M41	Proteobacteria	Betaproteobacteria	Burkholderiales	Burkholderiaceae	Burkholderia	Burkholderia phenazinium (97.48%)
**4./KY681857**	A172	Amanita	fulva	A72	Proteobacteria	Gammaproteobacteria	Enterobacteriales	Enterobacteriaceae	unclassif.Enterobacteriaceae	unclassif.Enterobacteriaceae
**10./KY681855**	A121	Amanita	fulva	K21	Proteobacteria	Gammaproteobacteria	Enterobacteriales	Enterobacteriaceae	unclassif.Enterobacteriaceae	unclassif.Enterobacteriaceae
**C_16b. 1492R/ KY681877**	A641	Amanita	fulva	M41	Proteobacteria	Gammaproteobacteria	Enterobacteriales	Enterobacteriaceae	unclassif.Enterobacteriaceae	unclassif.Enterobacteriaceae
**20./KY681876**	A333	Amanita	fulva	M33	Proteobacteria	Gammaproteobacteria	Enterobacteriales	Enterobacteriaceae	unclassif.Enterobacteriaceae	unclassif.Enterobacteriaceae
**25. 1492R / KY681822**	M33-A1	Amanita	fulva	M33	Proteobacteria	Gammaproteobacteria	Enterobacteriales	Enterobacteriaceae	unclassif.Enterobacteriaceae	unclassif.Enterobacteriaceae
**26./KY681866**	A372	Amanita	fulva	A72	Proteobacteria	Gammaproteobacteria	Enterobacteriales	Enterobacteriaceae	unclassif.Enterobacteriaceae	unclassif.Enterobacteriaceae
**27./KY681869**	A441	Amanita	fulva	M41	Proteobacteria	Gammaproteobacteria	Enterobacteriales	Enterobacteriaceae	unclassif.Enterobacteriaceae	unclassif.Enterobacteriaceae

**35./KY681875**	M33-A6	Amanita	fulva	M33	Proteobacteria	Gammaproteobacteria	Enterobacteriales	Enterobacteriaceae	unclassif.Enterobacteriaceae	unclassif.Enterobacteriaceae
**41./KY681837**	S317	Suillus	variegatus	K17	Proteobacteria	Gammaproteobacteria	Enterobacteriales	Enterobacteriaceae	unclassif.Enterobacteriaceae	unclassif.Enterobacteriaceae
**42./KY681872**	M33-A5	Amanita	fulva	M33	Proteobacteria	Gammaproteobacteria	Enterobacteriales	Enterobacteriaceae	unclassif.Enterobacteriaceae	unclassif.Enterobacteriaceae
**C_44b. 1492R*/ KY681878**	A272	Amanita	fulva	A72	Proteobacteria	Gammaproteobacteria	Enterobacteriales	Enterobacteriaceae	unclassif.Enterobacteriaceae	unclassif.Enterobacteriaceae
**49./KY681826**	M13-S5	Suillus	bovinus	M13	Proteobacteria	Gammaproteobacteria	Enterobacteriales	Enterobacteriaceae	unclassif.Enterobacteriaceae	unclassif.Enterobacteriaceae
**50./KY681824**	M13-S6	Suillus	bovinus	M13	Proteobacteria	Gammaproteobacteria	Enterobacteriales	Enterobacteriaceae	unclassif.Enterobacteriaceae	unclassif.Enterobacteriaceae
**51./KY681825**	M13-S3	Suillus	bovinus	M13	Proteobacteria	Gammaproteobacteria	Enterobacteriales	Enterobacteriaceae	unclassif.Enterobacteriaceae	unclassif.Enterobacteriaceae
**53./KY681828**	M13-S1	Suillus	bovinus	M13	Proteobacteria	Gammaproteobacteria	Enterobacteriales	Enterobacteriaceae	unclassif.Enterobacteriaceae	unclassif.Enterobacteriaceae
**57./KY681827**	M13-S4	Suillus	bovinus	M13	Proteobacteria	Gammaproteobacteria	Enterobacteriales	Enterobacteriaceae	unclassif.Enterobacteriaceae	unclassif.Enterobacteriaceae
**59./KY681860**	L119	Lactarius	rufus	K19	Proteobacteria	Gammaproteobacteria	Enterobacteriales	Enterobacteriaceae	unclassif.Enterobacteriaceae	unclassif.Enterobacteriaceae
**62./KY681868**	M13-S7	Suillus	bovinus	M13	Proteobacteria	Gammaproteobacteria	Enterobacteriales	Enterobacteriaceae	unclassif.Enterobacteriaceae	unclassif.Enterobacteriaceae
**64./KY681858**	M13-S2	Suillus	bovinus	M13	Proteobacteria	Gammaproteobacteria	Enterobacteriales	Enterobacteriaceae	unclassif.Enterobacteriaceae	unclassif.Enterobacteriaceae
**65./KY681829**	S319	Suillus	bovinus	K19	Proteobacteria	Gammaproteobacteria	Enterobacteriales	Enterobacteriaceae	unclassif.Enterobacteriaceae	unclassif.Enterobacteriaceae
**68./KY681856**	S219	Suillus	variegatus	K19	Proteobacteria	Gammaproteobacteria	Enterobacteriales	Enterobacteriaceae	unclassif.Enterobacteriaceae	unclassif.Enterobacteriaceae
**C_70b. 1492R*/ KY681879**	L341	Lactarius	rufus	M41	Proteobacteria	Gammaproteobacteria	Enterobacteriales	Enterobacteriaceae	unclassif.Enterobacteriaceae	unclassif.Enterobacteriaceae
**75./KY681838**	L241	Lactarius	rufus	M41	Proteobacteria	Gammaproteobacteria	Enterobacteriales	Enterobacteriaceae	unclassif.Enterobacteriaceae	unclassif.Enterobacteriaceae
**101./KY681859**	R219	Russula	paludosa	K19	Proteobacteria	Gammaproteobacteria	Enterobacteriales	Enterobacteriaceae	unclassif.Enterobacteriaceae	unclassif.Enterobacteriaceae
**116./KY681873**	R221	Russula	vinosa	K21	Proteobacteria	Gammaproteobacteria	Enterobacteriales	Enterobacteriaceae	unclassif.Enterobacteriaceae	unclassif.Enterobacteriaceae
**121./KY681870**	R139	Russula	vinosa	A39	Proteobacteria	Gammaproteobacteria	Enterobacteriales	Enterobacteriaceae	unclassif.Enterobacteriaceae	unclassif.Enterobacteriaceae
**126./KY681871**	R161	Russula	paludosa	A61	Proteobacteria	Gammaproteobacteria	Enterobacteriales	Enterobacteriaceae	unclassif.Enterobacteriaceae	unclassif.Enterobacteriaceae
**133./KY681839**	S372	Suillus	variegatus	A72	Proteobacteria	Gammaproteobacteria	Enterobacteriales	Enterobacteriaceae	unclassif.Enterobacteriaceae	unclassif.Enterobacteriaceae
**C_139. 1492R/ KY681956**	M13-1	Lactarius	rufus	M13	Proteobacteria	Gammaproteobacteria	Enterobacteriales	Enterobacteriaceae	unclassif.Enterobacteriaceae	unclassif.Enterobacteriaceae
**148. 27F/ KY681823**	S121	Suillus	variegatus	K21	Proteobacteria	Gammaproteobacteria	Enterobacteriales	Enterobacteriaceae	unclassif.Enterobacteriaceae	unclassif.Enterobacteriaceae
**167. 1492R/ KY681841**	L.holop.2/M14	Leccinum	holopus	M14	Proteobacteria	Gammaproteobacteria	Enterobacteriales	Enterobacteriaceae	unclassif.Enterobacteriaceae	unclassif.Enterobacteriaceae
**171. 1492R / KY681832**	S.bov.15/M33	Suillus	bovinus	M33	Proteobacteria	Gammaproteobacteria	Enterobacteriales	Enterobacteriaceae	unclassif.Enterobacteriaceae	unclassif.Enterobacteriaceae
**172. 1492R/ KY681834**	R.dec.50/M41	Russula	decolorans	M41	Proteobacteria	Gammaproteobacteria	Enterobacteriales	Enterobacteriaceae	unclassif.Enterobacteriaceae	unclassif.Enterobacteriaceae
**173. 1492R/ KY681835**	R.dec.50/M41	Russula	decolorans	M41	Proteobacteria	Gammaproteobacteria	Enterobacteriales	Enterobacteriaceae	unclassif.Enterobacteriaceae	unclassif.Enterobacteriaceae
**180. 1492R/ KY681847**	S.bov.16/M33	Suillus	bovinus	M33	Proteobacteria	Gammaproteobacteria	Enterobacteriales	Enterobacteriaceae	unclassif.Enterobacteriaceae	unclassif.Enterobacteriaceae
**181. 1492R/ KY681864**	S.bov.16/M33	Suillus	bovinus	M33	Proteobacteria	Gammaproteobacteria	Enterobacteriales	Enterobacteriaceae	unclassif.Enterobacteriaceae	unclassif.Enterobacteriaceae
**183. 1492R/ KY681853**	Cort.cap.2/M33	Cortinarius	caperatus	M33	Proteobacteria	Gammaproteobacteria	Enterobacteriales	Enterobacteriaceae	unclassif.Enterobacteriaceae	unclassif.Enterobacteriaceae
**186. 1492R/**	S.bov.1/M45	Suillus	bovinus	M45	Proteobacteria	Gammaproteobacteria	Enterobacteriales	Enterobacteriaceae	unclassif.Enterobacteriaceae	unclassif.Enterobacteriaceae

**KY681845**										
**189. 1492R / KY681821**	R.dec.1/M45	Russula	decolorans	M45	Proteobacteria	Gammaproteobacteria	Enterobacteriales	Enterobacteriaceae	unclassif.Enterobacteriaceae	unclassif.Enterobacteriaceae
**199. 1492R/ KY681863**	R.dec..2/M45	Russula	decolorans	M45	Proteobacteria	Gammaproteobacteria	Enterobacteriales	Enterobacteriaceae	unclassif.Enterobacteriaceae	unclassif.Enterobacteriaceae
**202. 1492R / KY681830**	S.bov.17/M33	Suillus	bovinus	M33	Proteobacteria	Gammaproteobacteria	Enterobacteriales	Enterobacteriaceae	unclassif.Enterobacteriaceae	unclassif.Enterobacteriaceae
**215. 1492R/ KY681854**	Cort.cap.1/M33	Cortinarius	caperatus	M33	Proteobacteria	Gammaproteobacteria	Enterobacteriales	Enterobacteriaceae	unclassif.Enterobacteriaceae	unclassif.Enterobacteriaceae
**218. 1492R/ KY681833**	Cort. cap.4/M47	Cortinarius	caperatus	M47	Proteobacteria	Gammaproteobacteria	Enterobacteriales	Enterobacteriaceae	unclassif.Enterobacteriaceae	unclassif.Enterobacteriaceae
**220. 1492R/ KY681843**	P.invol.3/M33	Paxillus	involutus	M33	Proteobacteria	Gammaproteobacteria	Enterobacteriales	Enterobacteriaceae	unclassif.Enterobacteriaceae	unclassif.Enterobacteriaceae
**224. 1492R/ KY681862**	P.invol.1/M41	Paxillus	involutus	M41	Proteobacteria	Gammaproteobacteria	Enterobacteriales	Enterobacteriaceae	unclassif.Enterobacteriaceae	unclassif.Enterobacteriaceae
**226. 1492R/ KY681842**	P.invol.2/M33	Paxillus	involutus	M33	Proteobacteria	Gammaproteobacteria	Enterobacteriales	Enterobacteriaceae	unclassif.Enterobacteriaceae	unclassif.Enterobacteriaceae
**243. 1492R/ KY681836**	C.a.1/M47	Cortinarius	armillatus	M47	Proteobacteria	Gammaproteobacteria	Enterobacteriales	Enterobacteriaceae	unclassif.Enterobacteriaceae	unclassif.Enterobacteriaceae
**262. 1492R/ KY681861**	C.c.1/M41	Cantharellus	cibarius	M41	Proteobacteria	Gammaproteobacteria	Enterobacteriales	Enterobacteriaceae	unclassif.Enterobacteriaceae	unclassif.Enterobacteriaceae
**265. 1492R/ KY681867**	C.a.1/M41	Cortinarius	armillatus	M41	Proteobacteria	Gammaproteobacteria	Enterobacteriales	Enterobacteriaceae	unclassif.Enterobacteriaceae	unclassif.Enterobacteriaceae
**272. 1492R/ KY681840**	P.invol.3/M33	Paxillus	involutus	M33	Proteobacteria	Gammaproteobacteria	Enterobacteriales	Enterobacteriaceae	unclassif.Enterobacteriaceae	unclassif.Enterobacteriaceae
**288. 1492R/ KY681874**	C.c.2/M33	Cantharellus	cibarius	M33	Proteobacteria	Gammaproteobacteria	Enterobacteriales	Enterobacteriaceae	unclassif.Enterobacteriaceae	unclassif.Enterobacteriaceae
**300. 1492R/ KY681844**	S.v.16/M41	Suillus	variegatus	M41	Proteobacteria	Gammaproteobacteria	Enterobacteriales	Enterobacteriaceae	unclassif.Enterobacteriaceae	unclassif.Enterobacteriaceae
**301. 1492R/ KY681846**	L.holop.2/M41	Leccinum	holopus	M41	Proteobacteria	Gammaproteobacteria	Enterobacteriales	Enterobacteriaceae	unclassif.Enterobacteriaceae	unclassif.Enterobacteriaceae
**302. 1492R/ KY681831**	L.holop.21/M41	Leccinum	holopus	M41	Proteobacteria	Gammaproteobacteria	Enterobacteriales	Enterobacteriaceae	unclassif.Enterobacteriaceae	unclassif.Enterobacteriaceae
**303. 1492R/ KY681851**	L. holop.20/M33	Leccinum	holopus	M33	Proteobacteria	Gammaproteobacteria	Enterobacteriales	Enterobacteriaceae	unclassif.Enterobacteriaceae	unclassif.Enterobacteriaceae
**305. 1492R/ KY681850**	L. holop.22/M33	Leccinum	holopus	M33	Proteobacteria	Gammaproteobacteria	Enterobacteriales	Enterobacteriaceae	unclassif.Enterobacteriaceae	unclassif.Enterobacteriaceae
**307. 1492R/ KY681849**	L.holop.21/M33	Leccinum	holopus	M33	Proteobacteria	Gammaproteobacteria	Enterobacteriales	Enterobacteriaceae	unclassif.Enterobacteriaceae	unclassif.Enterobacteriaceae
**308. 1492R/ KY681848**	L. scabr.17/M33	Leccinum	scabrum	M33	Proteobacteria	Gammaproteobacteria	Enterobacteriales	Enterobacteriaceae	unclassif.Enterobacteriaceae	unclassif.Enterobacteriaceae
**310. 1492R/ KY681865**	L.holop.20/M41	Leccinum	holopus	M41	Proteobacteria	Gammaproteobacteria	Enterobacteriales	Enterobacteriaceae	unclassif.Enterobacteriaceae	unclassif.Enterobacteriaceae
**328. 1492R / KY681852**	L.holop.20/M33	Leccinum	holopus	M33	Proteobacteria	Gammaproteobacteria	Enterobacteriales	Enterobacteriaceae	unclassif.Enterobacteriaceae	unclassif.Enterobacteriaceae

**3./KY681890**	M33-A6	Amanita	fulva	M33	Proteobacteria	Gammaproteobacteria	Pseudomonadales	Pseudomonadaceae	Pseudomonas	Pseudomonas trivialis(99.86%)
**5./KY681900**	A272	Amanita	fulva	A72	Proteobacteria	Gammaproteobacteria	Pseudomonadales	Pseudomonadaceae	Pseudomonas	Pseudomonas brenneri (100.00%)
**6./KY681887**	A121	Amanita	fulva	K21	Proteobacteria	Gammaproteobacteria	Pseudomonadales	Pseudomonadaceae	Pseudomonas	Pseudomonas fluorescens (99.07%)
**7./KY681895**	M33-A5	Amanita	fulva	M33	Proteobacteria	Gammaproteobacteria	Pseudomonadales	Pseudomonadaceae	Pseudomonas	Pseudomonas fluorescens(99.79%)
**8./KY681941**	M33-A6	Amanita	fulva	M33	Proteobacteria	Gammaproteobacteria	Pseudomonadales	Pseudomonadaceae	Pseudomonas	Pseudomonas fragi (99.50%)
**9./KY681897**	S217	Suillus	variegatus	K17	Proteobacteria	Gammaproteobacteria	Pseudomonadales	Pseudomonadaceae	Pseudomonas	Pseudomonas tolaasii (99.71%)
**11./KY681881**	A321	Amanita	fulva	K21	Proteobacteria	Gammaproteobacteria	Pseudomonadales	Pseudomonadaceae	Pseudomonas	Pseudomonas fluorescens(99.14%)
**12./KY681889**	A272	Amanita	fulva	A72	Proteobacteria	Gammaproteobacteria	Pseudomonadales	Pseudomonadaceae	Pseudomonas	Pseudomonas tolaasii (99.79%)
**15./KY681940**	A321	Amanita	fulva	K21	Proteobacteria	Gammaproteobacteria	Pseudomonadales	Pseudomonadaceae	Pseudomonas	Pseudomonas fragi (99.43%)
**17./KY681899**	A641	Amanita	fulva	M41	Proteobacteria	Gammaproteobacteria	Pseudomonadales	Pseudomonadaceae	Pseudomonas	Pseudomonas brenneri (100.00%)
**21./KY681909**	A172	Amanita	fulva	A72	Proteobacteria	Gammaproteobacteria	Pseudomonadales	Pseudomonadaceae	Pseudomonas	Pseudomonas brenneri (99.86%)
**22./KY681904**	M33-A5	Amanita	fulva	M33	Proteobacteria	Gammaproteobacteria	Pseudomonadales	Pseudomonadaceae	Pseudomonas	Pseudomonas brenneri (100.00%)
**23./KY681907**	A541	Amanita	fulva	M41	Proteobacteria	Gammaproteobacteria	Pseudomonadales	Pseudomonadaceae	Pseudomonas	Pseudomonas brenneri (99.93%)
**24./KY681893**	A219	Amanita	fulva	K19	Proteobacteria	Gammaproteobacteria	Pseudomonadales	Pseudomonadaceae	Pseudomonas	Pseudomonas fluorescens (99.79%)
**28./KY681911**	M33-A7	Amanita	fulva	M33	Proteobacteria	Gammaproteobacteria	Pseudomonadales	Pseudomonadaceae	Pseudomonas	Pseudomonas brenneri (99.93%)
**29./KY681882**	A133	Amanita	fulva	M33	Proteobacteria	Gammaproteobacteria	Pseudomonadales	Pseudomonadaceae	Pseudomonas	Pseudomonas fluorescens (99.14%)
**31./KY681883**	A221	Amanita	fulva	K21	Proteobacteria	Gammaproteobacteria	Pseudomonadales	Pseudomonadaceae	Pseudomonas	Pseudomonas fluorescens /(99.14%)
**32./KY681945**	A219	Amanita	fulva	K19	Proteobacteria	Gammaproteobacteria	Pseudomonadales	Pseudomonadaceae	Pseudomonas	Pseudomonas koreensis (99.50%)
**36./KY681891**	A233	Amanita	fulva	M33	Proteobacteria	Gammaproteobacteria	Pseudomonadales	Pseudomonadaceae	Pseudomonas	Pseudomonas trivialis (99.86%)
**37./KY681892**	A219	Amanita	fulva	K19	Proteobacteria	Gammaproteobacteria	Pseudomonadales	Pseudomonadaceae	Pseudomonas	Pseudomonas lurida (99.79%)
**C_38b. 1492R*/ KY681880**	A319	Amanita	fulva	K19	Proteobacteria	Gammaproteobacteria	Pseudomonadales	Pseudomonadaceae	Pseudomonas	Pseudomonas brenneri (97.33%)
**40./KY681910**	M33-A2	Amanita	fulva	M33	Proteobacteria	Gammaproteobacteria	Pseudomonadales	Pseudomonadaceae	Pseudomonas	Pseudomonas brenneri (99.93%)
**43./KY681901**	M33-A6	Amanita	fulva	M33	Proteobacteria	Gammaproteobacteria	Pseudomonadales	Pseudomonadaceae	Pseudomonas	Pseudomonas brenneri /(100.00%)
**52./KY681914**	M13-S6	Suillus	bovinus	M13	Proteobacteria	Gammaproteobacteria	Pseudomonadales	Pseudomonadaceae	Pseudomonas	Pseudomonas fluorescens (99%)
**60./KY681944**	L219	Lactarius	rufus	K19	Proteobacteria	Gammaproteobacteria	Pseudomonadales	Pseudomonadaceae	Pseudomonas	Pseudomonas koreensis (99.50%)
**66./KY681942**	S219	Suillus	variegatus	K19	Proteobacteria	Gammaproteobacteria	Pseudomonadales	Pseudomonadaceae	Pseudomonas	Pseudomonas fragi (99.43%)

**69./KY681943**	S119	Suillus	variegatus	K19	Proteobacteria	Gammaproteobacteria	Pseudomonadales	Pseudomonadaceae	Pseudomonas	Pseudomonas fragi (99.43%)
**71./KY681908**	S119	Suillus	variegatus	K19	Proteobacteria	Gammaproteobacteria	Pseudomonadales	Pseudomonadaceae	Pseudomonas	Pseudomonas brenneri (100.00%)
**72./KY681896**	L141	Lactarius	rufus	M41	Proteobacteria	Gammaproteobacteria	Pseudomonadales	Pseudomonadaceae	Pseudomonas	Pseudomonas fluorescens (99.71%)
**74./KY681948**	L361	Lactarius	rufus	A61	Proteobacteria	Gammaproteobacteria	Pseudomonadales	Pseudomonadaceae	Pseudomonas	Pseudomonas koreensis (99.00%)
**76./KY681902**	L341	Lactarius	rufus	M41	Proteobacteria	Gammaproteobacteria	Pseudomonadales	Pseudomonadaceae	Pseudomonas	Pseudomonas brenneri (100.00%)
**78./KY681903**	L119	Lactarius	rufus	K19	Proteobacteria	Gammaproteobacteria	Pseudomonadales	Pseudomonadaceae	Pseudomonas	Pseudomonas brenneri (100.00%)
**99./KY681949**	R319	Russula	paludosa	K19	Proteobacteria	Gammaproteobacteria	Pseudomonadales	Pseudomonadaceae	Pseudomonas	Pseudomonas fluorescens (99.64%)
**100./KY681953**	L1233	Lactarius	rufus	M33	Proteobacteria	Gammaproteobacteria	Pseudomonadales	Pseudomonadaceae	Pseudomonas	Pseudomonas denitrificans (99.68%)
**102./KY681912**	L1333	Lactarius	rufus	M33	Proteobacteria	Gammaproteobacteria	Pseudomonadales	Pseudomonadaceae	Pseudomonas	Pseudomonas brenneri (99.93%)
**105./KY681947**	R321	Russula	vinosa	K21	Proteobacteria	Gammaproteobacteria	Pseudomonadales	Pseudomonadaceae	Pseudomonas	Pseudomonas fluorescens(99.07%)
**106./KY681905**	R313	Russula	paludosa	M13	Proteobacteria	Gammaproteobacteria	Pseudomonadales	Pseudomonadaceae	Pseudomonas	Pseudomonas brenneri (100.00%)
**108./KY681884**	R741	Russula	vinosa	M41	Proteobacteria	Gammaproteobacteria	Pseudomonadales	Pseudomonadaceae	Pseudomonas	Pseudomonas fluorescens (99.93%)
**109./KY681894**	S121	Suillus	variegatus	K21	Proteobacteria	Gammaproteobacteria	Pseudomonadales	Pseudomonadaceae	Pseudomonas	Pseudomonas fluorescens (99.14%)
**110./KY681886**	S221	Suillus	variegatus	K21	Proteobacteria	Gammaproteobacteria	Pseudomonadales	Pseudomonadaceae	Pseudomonas	Pseudomonas fluorescens(99.14%)
**115./KY681898**	R272	Russula	emetica	A72	Proteobacteria	Gammaproteobacteria	Pseudomonadales	Pseudomonadaceae	Pseudomonas	Pseudomonas tolaasii (99.71%)
**119. 1492R*/ KY682048**	R272	Russula	emetica	A72	Proteobacteria	Gammaproteobacteria	Pseudomonadales	Pseudomonadaceae	Pseudomonas (97.29%)	unclassif.Pseudomonas sp.
**127./KY681885**	R261	Russula	decolorans	A61	Proteobacteria	Gammaproteobacteria	Pseudomonadales	Pseudomonadaceae	Pseudomonas	Pseudomonas fluorescens (99.14%)
**129./KY681950**	R219	Russula	paludosa	K19	Proteobacteria	Gammaproteobacteria	Pseudomonadales	Pseudomonadaceae	Pseudomonas	Pseudomonas fluorescens (99.78%)
**131./KY681913**	A119	Amanita	fulva	K19	Proteobacteria	Gammaproteobacteria	Pseudomonadales	Pseudomonadaceae	Pseudomonas	Pseudomonas brenneri (99.93%)
**132./KY681952**	M33-1	Lactarius	rufus	M33	Proteobacteria	Gammaproteobacteria	Pseudomonadales	Pseudomonadaceae	Pseudomonas	Pseudomonas fluorescens (99.93%)
**165./KY681951**	M33-3	Lactarius	rufus	M33	Proteobacteria	Gammaproteobacteria	Pseudomonadales	Pseudomonadaceae	Pseudomonas	Pseudomonas fluorescens(99.93%)
**168./KY681906**	A117	Amanita	muscaria	K17	Proteobacteria	Gammaproteobacteria	Pseudomonadales	Pseudomonadaceae	Pseudomonas	Pseudomonas brenneri (100.00%)
**185. 1492R/ KY681930**	A.f.1/M47	Amanita	fulva	M47	Proteobacteria	Gammaproteobacteria	Pseudomonadales	Pseudomonadaceae	Pseudomonas	Pseudomonas fluorescens (99.65%)

**190. 1492R / KY681888**	R.dec.15/M47	Russula	decolorans	M47	Proteobacteria	Gammaproteobacteria	Pseudomonadales	Pseudomonadaceae	Pseudomonas	Pseudomonas fluorescens (99.04%)
**196. 1492R/ KY681946**	R.dec.62/M14	Russula	decolorans	M14	Proteobacteria	Gammaproteobacteria	Pseudomonadales	Pseudomonadaceae	Pseudomonas	Pseudomonas veronii (99.36%)
**211. 1492R/ KY681933**	A.f.2/M47	Amanita	fulva	M47	Proteobacteria	Gammaproteobacteria	Pseudomonadales	Pseudomonadaceae	Pseudomonas	Pseudomonas veronii (99.56%)
**212. 1492R/ KY681921**	A.f.3/M47	Amanita	fulva	M47	Proteobacteria	Gammaproteobacteria	Pseudomonadales	Pseudomonadaceae	Pseudomonas	Pseudomonas brenneri (99.39%)
**216. 1492R/ KY681928**	Cort.cap.1/M33	Cortinarius	caperatus	M33	Proteobacteria	Gammaproteobacteria	Pseudomonadales	Pseudomonadaceae	Pseudomonas	Pseudomonas tolaasii(99.56%)
**235. 1492R/ KY681954**	C.a.2/M41	Cortinarius	armillatus	M41	Proteobacteria	Gammaproteobacteria	Pseudomonadales	Pseudomonadaceae	Pseudomonas	Pseudomonas fluorescens (98.45%)
**237. 1492R/ KY681916**	C.a.2/M41	Cortinarius	armillatus	M41	Proteobacteria	Gammaproteobacteria	Pseudomonadales	Pseudomonadaceae	Pseudomonas	Pseudomonas brenneri (99.74%)
**242. 1492R/ KY681915**	C.a.1/M47	Cortinarius	armillatus	M47	Proteobacteria	Gammaproteobacteria	Pseudomonadales	Pseudomonadaceae	Pseudomonas	Pseudomonas brenneri (99.65%)
**244. 1492R/ KY681932**	C.a.2/M47	Cortinarius	armillatus	M47	Proteobacteria	Gammaproteobacteria	Pseudomonadales	Pseudomonadaceae	Pseudomonas	Pseudomonas veronii (99.56%)
**246. 1492R/ KY681920**	C.a.2/M13	Cortinarius	armillatus	M13	Proteobacteria	Gammaproteobacteria	Pseudomonadales	Pseudomonadaceae	Pseudomonas	Pseudomonas brenneri (99.92%)
**255. 1492R/ KY681924**	C.c.3/M47	Cantharellus	cibarius	M47	Proteobacteria	Gammaproteobacteria	Pseudomonadales	Pseudomonadaceae	Pseudomonas	Pseudomonas migulae (99.48%)
**258. 1492R/ KY681934**	C.c.2/M45	Cantharellus	cibarius	M45	Proteobacteria	Gammaproteobacteria	Pseudomonadales	Pseudomonadaceae	Pseudomonas	Pseudomonas veronii (99.65%)
**261. 1492R/ KY681925**	C.c.4/M47	Cantharellus	cibarius	M47	Proteobacteria	Gammaproteobacteria	Pseudomonadales	Pseudomonadaceae	Pseudomonas	Pseudomonas migulae (99.75%)
**264. 1492R/ KY681939**	C.c.1/M41	Cantharellus	cibarius	M41	Proteobacteria	Gammaproteobacteria	Pseudomonadales	Pseudomonadaceae	Pseudomonas	Pseudomonas fluorescens (99.33%)
**269. 1492R/ KY681922**	C.c.3/M13	Cantharellus	cibarius	M13	Proteobacteria	Gammaproteobacteria	Pseudomonadales	Pseudomonadaceae	Pseudomonas	Pseudomonas brenneri (99.48%)
**275. 1492R/ KY681937**	C.c.1/M33	Cantharellus	cibarius	M33	Proteobacteria	Gammaproteobacteria	Pseudomonadales	Pseudomonadaceae	Pseudomonas	Pseudomonas fluorescens (98.44%)
**277. 1492R/ KY681936**	C.c.3/M33	Cantharellus	cibarius	M33	Proteobacteria	Gammaproteobacteria	Pseudomonadales	Pseudomonadaceae	Pseudomonas	Pseudomonas veronii (99.56%)
**278. 1492R/ KY681919**	C.c.3/M33	Cantharellus	cibarius	M33	Proteobacteria	Gammaproteobacteria	Pseudomonadales	Pseudomonadaceae	Pseudomonas	Pseudomonas brenneri (99.74%)
**290. 1492R/ KY681926**	C.c.2/M33	Cantharellus	cibarius	M33	Proteobacteria	Gammaproteobacteria	Pseudomonadales	Pseudomonadaceae	Pseudomonas	Pseudomonas migulae (99.58%)
**292. 1492R/ KY681955**	C.c.2/M41	Cantharellus	cibarius	M41	Proteobacteria	Gammaproteobacteria	Pseudomonadales	Pseudomonadaceae	Pseudomonas	Pseudomonas lini (97.94%)
**294. 1492R/ KY681923**	L. variic.18/M33	Leccinum	variicolor	M33	Proteobacteria	Gammaproteobacteria	Pseudomonadales	Pseudomonadaceae	Pseudomonas	Pseudomonas brenneri (99.48%)
**297. 1492R/**	C.c.3/M47	Cantharellus	cibarius	M47	Proteobacteria	Gammaproteobacteria	Pseudomonadales	Pseudomonadaceae	Pseudomonas	Pseudomonas veronii (99.65%)

**KY681935**										
**298. 1492R/ KY681918**	C.c.1/M45	Cantharellus	cibarius	M45	Proteobacteria	Gammaproteobacteria	Pseudomonadales	Pseudomonadaceae	Pseudomonas	Pseudomonas brenneri (99.74%)
**299. 1492R/ KY681929**	P. invol.3/M33	Paxillus	involutus	M33	Proteobacteria	Gammaproteobacteria	Pseudomonadales	Pseudomonadaceae	Pseudomonas	Pseudomonas fluorescens (99.74%)
**306. 1492R/ KY681917**	C.c.1/M33	Cantharellus	cibarius	M33	Proteobacteria	Gammaproteobacteria	Pseudomonadales	Pseudomonadaceae	Pseudomonas	Pseudomonas brenneri (99.74%)
**317. 1492R/ KY681927**	L.r.1/M47	Lactarius	rufus	M47	Proteobacteria	Gammaproteobacteria	Pseudomonadales	Pseudomonadaceae	Pseudomonas	Pseudomonas tolaasii (99.56%)
**319. 1492R/ KY681938**	C.c.1/M45	Cantharellus	cibarius	M45	Proteobacteria	Gammaproteobacteria	Pseudomonadales	Pseudomonadaceae	Pseudomonas	Pseudomonas fluorescens (99.39%)
**326. 1492R/ KY681931**	C.c.1/M47	Cantharellus	cibarius	M47	Proteobacteria	Gammaproteobacteria	Pseudomonadales	Pseudomonadaceae	Pseudomonas	Pseudomonas veronii (99.56%)
**14./KY682054**	A319	Amanita	fulva	K19	Proteobacteria	Gammaproteobacteria	Xanthomonadales	Xanthomonadaceae	Stenotrophomonas	Stenotrophomonas rhizophila (99.93%)
**90./KY682049**	L317	Lactarius	rufus	K17	Proteobacteria	Gammaproteobacteria	Xanthomonadales	Xanthomonadaceae	Luteibacter	Luteibacter rhizovicinus (99.86%)
**91./KY682050**	L117	Lactarius	rufus	K17	Proteobacteria	Gammaproteobacteria	Xanthomonadales	Xanthomonadaceae	Luteibacter	Luteibacter rhizovicinus (99.86%)
**93.*/KY682052**	L317	Lactarius	rufus	K17	Proteobacteria	Gammaproteobacteria	Xanthomonadales	Xanthomonadaceae	Dyella	Dyella japonica (99.72%)
**117./KY682055**	R172	Russula	emetica	A72	Proteobacteria	Gammaproteobacteria	Xanthomonadales	Xanthomonadaceae	Stenotrophomonas	Stenotrophomonas rhizophila (99.93%)
**123./KY682056**	R321	Russula	vinosa	K21	Proteobacteria	Gammaproteobacteria	Xanthomonadales	Xanthomonadaceae	Stenotrophomonas	Stenotrophomonas rhizophila (99.93%)
**150.*/KY682053**	R121	Russula	rhodopus	K21	Proteobacteria	Gammaproteobacteria	Xanthomonadales	Xanthomonadaceae	Dyella	Dyella marensis (99.72%)
**201. 1492R/ KY682051**	R.dec.16/M47	Russula	decolorans	M47	Proteobacteria	Gammaproteobacteria	Xanthomonadales	Xanthomonadaceae	Luteibacter	Luteibacter rhizovicinus (99.73%)

*isolates representing OTUs that were not detected by HTS

Table S4. Number of PCR cycles for each sample.

Sample no	GenBank accession numbers	Fruitbody code	Fungal species	PCR cycles
**49.**	SAMN06618440	S.v.15/M13	*Suillus variegatus*	30
**50.**	SAMN06618441	S.bov.1/M45	*Suillus bovinus*	30
**51.**	SAMN06618442	Cort.cap.2/M45	*Cortinarius caperatus*	30
**52.**	SAMN06618443	C.c.3/M47	*Cantharellus cibarius*	25
**53.**	SAMN06618444	A.f.71/M33	*Amanita fulva*	30
**54.**	SAMN06618445	C.a.3/M33	*Cortinarius armillatus*	33
**55.**	SAMN06618446	Cort.cap.1/M47	*Cortinarius caperatus*	30
**56.**	SAMN06618447	C.c.3/M13	*Cantharellus cibarius*	28
**57.**	SAMN06618448	Cort.cap.1/M45	*Cortinarius caperatus*	30
**58.**	SAMN06618449	C.c.2/M47	*Cantharellus cibarius*	30
**59.**	SAMN06618450	Cort.cap.1/M47	*Cortinarius caperatus*	30
**60.**	SAMN06618451	C.a.2/M33	*Cortinarius armillatus*	33
**61.**	SAMN06618452	L.scabr.1/M47	*Leccinum scabrum*	33
**63.**	SAMN06618453	L.variic.18/M33	*Leccinum variicolor*	33
**64.**	SAMN06618454	P.invol.1/M13	*Paxillus involutus*	33
**65.**	SAMN06618455	S.v.15/M41	*Suillus variegatus*	28
**66.**	SAMN06618456	Cort.cap.3/M47	*Cortinarius caperatus*	30
**67.**	SAMN06618457	L.scabr.1/M41	*Leccinum scabrum*	33
**68.**	SAMN06618458	L.scabr.2/M47	*Leccinum scabrum*	30
**69.**	SAMN06618459	S.v.1/M47	*Suillus variegatus*	30
**70.**	SAMN06618460	A.f.2/M47	*Amanita fulva*	30
**71.**	SAMN06618461	S.v.1/M45	*Suillus variegatus*	33
**72.**	SAMN06618462	S.v.2/M47	*Suillus variegatus*	33
**73.**	SAMN06618463	P.invol.2/M13	*Paxillus involutus*	33
**74.**	SAMN06618464	C.c.4/M47	*Cantharellus cibarius*	28
**75.**	SAMN06618465	S.bov.2/M45	*Suillus bovinus*	35
**76.**	SAMN06618466	L.variic.19/M33	*Leccinum variicolor*	35
**77.**	SAMN06618467	Cort.cap.4/M47	*Cortinarius caperatus*	30
**78.**	SAMN06618468	S.bov.4/M45	*Suillus bovinus*	30
**79.**	SAMN06618469	P.invol.3/M13	*Paxillus involutus*	33
**80.**	SAMN06618470	S.v.17/M13	*Suillus variegatus*	33
**81.**	SAMN06618471	S.v.18/M13	*Suillus variegatus*	30
**82.**	SAMN06618472	S.bov.3/M45	*Suillus bovinus*	30
**83.**	SAMN06618473	A.f.66/M14	*Amanita fulva*	30
**84.**	SAMN06618474	P.invol.2/M41	*Paxillus involutus*	35
**85.**	SAMN06618475	R.dec.16/M47	*Russula decolorans*	30
**86.**	SAMN06618476	S.bov.16/M13	*Suillus bovinus*	30
**87.**	SAMN06618477	A.f.1/M47	*Amanita fulva*	30
**88.**	SAMN06618478	L.variic.15/M33	*Leccinum variicolor*	35
**89.**	SAMN06618479	P.invol.1/M41	*Paxillus involutus*	28
**90.**	SAMN06618480	R.dec.15/M47	*Russula decolorans*	30

**91.**	SAMN06618481	R.dec.51/M41	*Russula decolorans*	30
**92.**	SAMN06618482	S.bov.15/M13	*Suillus bovinus*	30
**93.**	SAMN06618483	L.variic.16/M33	*Leccinum variicolor*	35
**94.**	SAMN06618484	R.dec.60/M14	*Russula decolorans*	35
**95.**	SAMN06618485	Cort.cap.2/M47	*Cortinarius caperatus*	30
**96.**	SAMN06618486	L.scabr.5/M47	*Leccinum scabrum*	30
**97.**	SAMN06618487	M13-C4	*Cortinarius caperatus*	30
**98.**	SAMN06618488	M13-C2	*Cortinarius caperatus*	30
**99.**	SAMN06618489	S.bov.17/M13	*Suillus bovinus*	30
**100.**	SAMN06618490	S.v.3/M47	*Suillus variegatus*	30
**101.**	SAMN06618491	Cort.cap.75/M13	*Cortinarius caperatus*	30
**102.**	SAMN06618492	R.dec.17/M47	*Russula decolorans*	30
**103.**	SAMN06618493	P.invol.3/M41	*Paxillus involutus*	35
**104.**	SAMN06618494	R.dec.52/M41	*Russula decolorans*	30
**105.**	SAMN06618495	L.scabr.17/M33	*Leccinum scabrum*	28
**106.**	SAMN06618496	A.f.3/M47	*Amanita fulva*	30
**107.**	SAMN06618497	R.dec.62/M14	*Russula decolorans*	30
**108.**	SAMN06618498	S.v.16/M41	*Suillus variegatus*	30
**109.**	SAMN06618499	R.dec.61/M14	*Russula decolorans*	30
**110.**	SAMN06618500	P.invol.3/M33	*Paxillus involutus*	28
**111.**	SAMN06618501	P.invol.1/M33	*Paxillus involutus*	28
**112.**	SAMN06618502	C.a.2/M47	*Cortinarius armillatus*	35
**113.**	SAMN06618503	P.invol.2/M33	*Paxillus involutus*	28
**114.**	SAMN06618504	C.a.1/M47	*Cortinarius armillatus*	35
**115.**	SAMN06618505	L.scabr.2/M41	*Leccinum scabrum*	35
**116.**	SAMN06618506	Cort.cap.2/M45	*Cortinarius caperatus*	30
**117.**	SAMN06618507	C.a.1/M33	*Cortinarius armillatus*	35
**118.**	SAMN06618508	L.scabr.3/M47	*Leccinum scabrum*	28
**119.**	SAMN06618509	C.c.1/M33	*Cantharellus cibarius*	28
**120.**	SAMN06618510	Cort.cap.1/M33	*Cortinarius caperatus*	28
**121.**	SAMN06618511	C.c.3/M33	*Cantharellus cibarius*	28
**122.**	SAMN06618512	C.a.2/M13	*Cortinarius caperatus*	35
**123.**	SAMN06618513	C.a.1/M13	*Cortinarius armillatus*	35
**124.**	SAMN06618514	A.f.15/M14	*Amanita fulva*	30
**125.**	SAMN06618515	C.c.2/M33	*Cantharellus cibarius*	28
**126.**	SAMN06618516	C.c.1/M47	*Cantharellus cibarius*	28
**127.**	SAMN06618517	R.dec.2/M45	*Russula decolorans*	35
**128.**	SAMN06618518	R.dec.1/M45	*Russula decolorans*	35
**129.**	SAMN06618519	C.a.3/M41	*Cortinarius armillatus*	35
**130.**	SAMN06618520	R.dec.50/M41	*Russula decolorans*	33
**131.**	SAMN06618521	C.c.2/M45	*Cantharellus cibarius*	28
**132.**	SAMN06618522	C.a.2/M41	*Cortinarius armillatus*	35
**133.**	SAMN06618523	C.a.1/M41	*Cortinarius armillatus*	35
**134.**	SAMN06618524	C.c.1/M45	*Cantharellus cibarius*	28
**135.**	SAMN06618525	C.c.2/M41	*Cantharellus cibarius*	28
**136.**	SAMN06618526	C.c.1/M41	*Cantharellus cibarius*	28

**137.**	SAMN06618527	L.holop.1/M14	*Leccinum holopus*	30
**138.**	SAMN06618528	S.bov.17/M33	*Suillus bovinus*	30
**139.**	SAMN06618529	L.variic.1/M14	*Leccinum variicolor*	30
**140.**	SAMN06618530	L.holop.2/M14	*Leccinum holopus*	33
**142.**	SAMN06618531	L.variic.2/M14	*Leccinum variicolor*	30
**143.**	SAMN06618532	Cort.cap.3/M33	*Cortinarius caperatus*	30
**144.**	SAMN06618533	L.holop.21/M33	*Leccinum holopus*	33
**146.**	SAMN06618534	L.holop.20/M41	*Leccinum holopus*	28
**147.**	SAMN06618535	S.bov.16/M33	*Suillus bovinus*	33
**148.**	SAMN06618536	S.bov.15/M33	*Suillus bovinus*	30
**149.**	SAMN06618537	L.holop.20/M33	*Leccinum holopus*	33
**150.**	SAMN06618538	Cort.cap.2/M33	*Cortinarius caperatus*	30
**ME14**	SAMN06618539	ME14	soil	28
**ME45**	SAMN06618540	ME45	soil	28
**ME47**	SAMN06618541	ME47	soil	25
**154.**	SAMN06618542	L.scabr.18/M33	*Leccinum scabrum*	35
**B1**	SAMN06618543	L119, L219, L319	*Lactarius rufus*	33
**B10**	SAMN06618544	L141, L241, L341	*Lactarius rufus*	33
**B11**	SAMN06618545	L113, L213, L313	*Lactarius rufus*	33
**B12**	SAMN06618546	L172, L272,L372	*Lactarius quieticolor*	33
**B13**	SAMN06618547	L161, L261, L361	*Lactarius rufus*	33
**B14**	SAMN06618548	L139, L239, L339	*Lactarius rufus*	30
**B16**	SAMN06618549	S161, S261, S361	*Suillus variegatus*	35
**B17**	SAMN06618550	S139, S239, S339	*Suillus bovinus*	33
**B18**	SAMN06618551	A172, A272, A372	*Amanita fulva*	30
**B19**	SAMN06618552	M13-S2, M13-S3, M13-S5	*Suillus bovinus*	30
**B2**	SAMN06618553	L121, L221, L321	*Lactarius rufus*	33
**B20**	SAMN06618554	A.f.16,17,18/M41	*Amanita fulva*	30
**B21**	SAMN06618555	R119, R219, R319	*Russula sp.*	33
**B22**	SAMN06618556	R121, R221, R321	*Russula sp.*	30
**B23**	SAMN06618557	R117, R217, R317	*Russula sp.*	33
**B24**	SAMN06618558	R.emet1,2,3/A72	*Russula sp.*	30
**B25**	SAMN06618559	R161, R261, R361	*Russula sp.*	30
**B26**	SAMN06618560	R139, R239, R339	*Russula sp.*	33
**B27**	SAMN06618561	R.dec.16,17,18/M41	*Russula decolorans*	33
**B28**	SAMN06618562	L.r.D3/M13	*Lactarius rufus*	30
**B29**	SAMN06618563	S319	*Suillus bovinus*	30
**B3**	SAMN06618564	L117, L217, L317	*Lactarius rufus*	33
**B4**	SAMN06618565	S119,S219	*Suillus variegatus*	30
**B5**	SAMN06618566	S121, S221	*Suillus variegatus*	30
**B6**	SAMN06618567	S117, S217, S317	*Suillus variegatus*	30
**B7**	SAMN06618568	A119, A219, A319	*Amanita fulva*	30
**B8**	SAMN06618569	A121, A221, A321	*Amanita fulva*	30
**B9**	SAMN06618570	L1133, L1233, L1333	*Lactarius rufus*	30

**A39**	SAMN06618571	A39	soil	28
**A61**	SAMN06618572	A61	soil	28
**A72**	SAMN06618573	A72	soil	28
**K17**	SAMN06618574	K17	soil	28
**K19**	SAMN06618575	K19	soil	28
**K21**	SAMN06618576	K21	soil	28
**ME13**	SAMN06618577	ME13	soil	28
**ME33**	SAMN06618578	ME33	soil	28
**ME41**	SAMN06618579	ME41	soil	28

Table S5. Dominant bacterial taxa in fungal fruitbodies detected using HTS and culturing.

Phylum	Class	Order	Family/Genus	Cantharellus (12[a]/12[b])	Lactarius (28/41)	Russula (32/32)	Suillus (38/33)	Paxillus (9/5)	Leccinum (19/14)	Cortinarius (24/15)	Amanita (18/25)	In total (180/177)
**PROTEO-BACTERIA**				46.4*/100**	94.7/100	77.1/91	83.4/97	80.1/100	53.6/100	46.1/93	99.0/100	55.1/97
	GAMMA-PROTEOBACTERIA			8.1/100	8.9/34	41.2/59	60.0/64	8.8/60	13.4/64	22.2/53	90.7/96	17.7/62
		**Pseudomonadales**		0.4/92	0.2/24	24.3/28	37.7/18	7.5/20	9.5/7	18.1/33	77.3/80	8.7/36
			Pseudomonadaceae	0.4/92	0.1/24	14.7/28	36.9/18	6.9/20	4.5/7	11.0/33	77.2/80	8.1/36
			Pseudomonas	0.4/92	0.1/24	14.7/28	36.9/18	6.9/20	4.5/7	11.0/33	77.2/80	8.1/36
			Moraxellaceae	-/-	0.1/-	9.6/-	0.7/-	0.6/-	5.0/-	7.0/-	0.1/-	0.6/-
		**Enterobacteriales**		-/33	0.6/10	14.9/25	21.4/52	0.4/60	1.6/57	0.2/33	13.3/40	2.9/33
			Enterobacteriaceae	-/33	0.6/10	14.9/25	21.4/52	0.4/60	1.6/57	0.2/33	13.3/40	2.9/33
		**Legionellales**		4.9/-	-/-	-/-	0.3/-	0.2/-	0.1/-	1.5/-	-/-	3.7/-
			Coxiellaceae	4.0/-	-/-	-/-	0.2/-	-/-	-/-	-/-	-/-	3.0/-
			Aquicella	3.4/-	-/-	-/-	0.2/-	-/-	-/-	-/-	-/-	2.5/-
		**Xanthomonadales**		0.8/-	5.3/5	0.4/13	0.4/-	0.4/-	0.9/-	0.3/-	-/4	0.7/4
			Xanthomonadaceae	0.8/-	5.1/5	0.4/13	0.1/-	0.3/-	0.8/-	0.3/-	-/4	0.7/4
			Luteibacter	0.8/-	-/5	-/3	-/-	-/-	0.7/-	-/-	-/-	0.6/2
	BETA-PROTEOBACTERIA			10.5/17	84.7/71	22.5/38	18.1/42	44.1/60	9.3/57	4.2/60	8.1/4	13.6/44
		**Burkholderiales**		10.5/17	84.7/71	21.6/38	17.8/42	43.8/60	5.8/57	3.4/60	8.1/4	13.4/44
			Burkholderiacea	1.4/8	84.5/68	9.7/34	17.0/42	43.3/60	2.1/57	0.9/60	2.0/4	5.9/42
			Burkholderia	1.4/8	84.5/68	9.7/31	17.0/42	43.3/60	1.9/57	0.8/60	2.0/4	5.9/42
			Oxalobacteriaceae	6.2/8	-/2	11.3/3	0.4/-	0.2/-	1.8/-	1.2/-	6.1/-	5.2/2
			Massilia	2.9/-	-/-	2.4/-	0.1/-	-/-	1.1/-	0.1/-	-/-	2.3/-
			Comamonadaceae	2.8/8	0.1/2	0.1/-	0.4/-	0.4/-	1.2/-	0.7/-	-/-	2.2/1
			Acidovorax	2.4/-	-/-	0.1/-	0.3/-	0.2/-	0.3/-	-/-	-/-	1.8/-

	ALPHA-PROTEOBACTERIA			27.3/-	1.1/2	13.4/9	4.1/-	26.8/-	29.4/-	16.6/-	0.2/-	23.2/2
		**Rhizobiales**		19.3/-	0.4/2	1.8/6	0.7/-	1.2/-	6.4/-	2.2/-	0.1/-	14.7/2
			Rhizobiaceae	15.9/-	-/2	1.1/3	0.2/-	0.1/-	-/-	0.1/-	-/-	11.8/1
			Rhizobium	15.9/-	-/2	1.1/3	0.1/-	0.1/-	-/-	0.1/-	-/-	11.8/1
			Bradyrhizobiaceae	2.7/-	-/-	0.1/-	-/-	0.2/-	0.2/-	0.4/-	-/-	2.0/-
		**Rhodospirillales**		3.9/-	0.5/-	0.3/-	1.7/-	0.2/-	3.1/-	3.1/-	-/-	3.3/-
			Rhodospirillaceae	3.7/-	0.5/-	0.2/-	0.3/-	-/-	2.5/-	2.1/-	-/-	2.9/-
			Telmatospirillum	3.5/-	0.5/-	0.1/-	-/-	-/-	0.4/-	-/-	-/-	2.9/-
		**Sphingomonadales**		3.3/-	0.1/-	3.3/3	1.0/-	4.0/-	6.7/-	10.3/-	0.1/-	3.2/0.6
			Sphingomonadaceae	3.3/-	0.1/-	3.3/3	1.0/-	4.0/-	6.7/-	9.5/-	0.1/-	3.2/0.6
			Sphingomonas	1.1/-	-/-	1.2/3	0.9/-	3.6/-	4.0/-	7.2/-	-/-	1.4/0.6
			Novosphingobium	2.2/-	-/-	1.7/-	-/-	-/-	1.3/-	0.2/-	-/-	1.7/-
**BACTEROIDETES**				46.7/8	2.6/-	14.7/-	8.4/-	12.6/-	23.7/-	31.7/-	0.3/8	37.4/2
	BACTEROIDIA			-/-	2.2/-	0.4/-	4.3/-	7.1/-	9.8/-	2.1/-	-/-	1.1/-
		**Bacteroidales**		-/-	2.2/-	0.4/-	4.3/-	7.1/-	9.8/-	2.1/-	-/-	1.1/-
			Bacteroidaceae	-/-	2.2/-	0.4/-	4.3/-	7.1/-	9.8/-	2.1/-	-/-	1.1/-
			Bacteroides	-/-	2.2/-	0.4/-	4.3/-	7.1/-	9.8/-	2.1/-	-/-	1.1/-
	CYTOPHAGIA			0.1/-	-/-	1.9/-	2.8/-	4.3/-	3.6/-	23.6/-	0.2/-	1.0/-
		**Cytophagales**		0.1/-	-/-	1.9/-	2.8/-	4.3/-	3.6/-	23.6/-	0.2/-	1.0/-
			Cytophagaceae	0.1/-	-/-	1.7/-	2.3/-	4.1/-	3.4/-	17.7/-	0.2/-	0.8/-
			Cytophaga	-/-	-/-	1.7/-	2.1/-	4.1/-	3.4/-	17.7/-	0.2/-	0.8/-

	SPHINGO-BACTERIIA			46.6/8	0.1/-	8.5/-	1.1/-	1.2/-	2.5/-	4.8/-	0.1/4	35/1
		**Sphingo-bacteriales**		46.6/8	0.1/-	8.5/-	1.1/-	1.2/-	2.5/-	4.8/-	0.1/4	35/1
			Sphingobacteriaceae	17.2/8	0.1/-	8.2/-	0.9/-	0.7/-	1.7/-	3.8/-	0.1/4	13.1/1
			Mucilaginibacter	3.9/-	-/-	6.7/-	0.5/-	-/-	-/-	0.6/-	-/-	3.1/-
			Pedobacter	12.4/-	0.1/-	1.5/-	0.4/-	0.5/-	0.4/-	2.7/-	0.1/-	9.3/-
			Chitinophagaceae	29.4/-	-/-	0.3/-	0.1/-	0.5/-	0.8/-	1.0/-	-/-	21.8/-
			Chitinophaga	22.6/-	-/-	0.1/-	-/-	0.1/-	0.2/-	0.1/-	-/-	16.7/-
**FIRMICUTES**				-/-	1.5/-	2.0/9	1.4/3	0.7/20	1.7/-	6.4/7	0.1/-	0.4/3
	BACILLI			-/-	1.2/-	1.9/9	1.3/3	0.4/20	1.0/-	4.9/7	0.1/-	0.3/3
		**Bacillales**		-/-	0.4/-	1.2/9	1.0/3	0.3/20	0.5/-	4.1/7	0.1/-	0.2/3
**ACIDOBACTERIA**				3.5/-	0.4/-	0.3/-	1.7/-	0.3/-	5.9/-	1.1/-	-/-	3.0/-
	ACIDOBACTERIIA			3.5/-	0.4/-	0.3/-	1.7/-	0.3/-	5.9/-	1.1/-	-/-	2.9/-
		**Acidobacteriales**		3.5/-	0.4/-	0.3/-	1.3/-	0.3/-	4.3/-	-/-	-/-	2.9/-
			Acidobacteriaceae	3.5/-	0.4/-	0.3/-	1.2/-	0.3/-	4.3/-	-/-	-/-	2.9/-
			Terriglobus	3.0/-	-/-	-/-	0.1/-	-/-	0.2/-	-/-	-/-	2.2/-
**ACTINOBACTERIA**				2.5/8	0.9/-	4.2/6	3.6/-	2.1/-	8.2/-	10.8/-	0.4/-	2.8/2
	ACTINOBACTERIA			2.5/8	0.9/-	4.2/6	3.3/-	1.8/-	6.3/-	10.6/-	0.4/-	2.7/2
		**Actinomycetales**		-/8	-/-	0.4/6	0.2/-	0.2/-	0.3/-	1.5/-	0.1/-	0.1/2
		**Micrococcales**		2.5/8	0.1/-	1.7/6	0.4/-	1.3/-	2.3/-	7.4/-	0.2/-	2.2/2
			Microbacteriaceae	2.5/8	-/-	1.1/6	0.2/-	1.1/-	1.8/-	5.9/-	-/-	2.1/2
			Frigoribacterium	2.3/-	-/-	0.5/-	0.2/-	0.5/-	0.7/-	2.4/-	-/-	1.8/-

**VERRUCO-MICROBIA**	0.7/-	-/-	-/-	0.4/-	-/-	0.8/-	0.1/-	-/-	0.6/-

*The first column in each cell shows the relative abundance of the bacterial taxon based on HTS read numbers normalized by the total number of reads in each fungal genus; **the second column represents the proportion of fruitbodies from which a particular bacterial taxon was isolated into culture divided by the total number of sampled fruitbodies in that fungal genus;

[a] the first column in each cell in the first row shows the number of fruitbodies analysed by HTS; [b] the second column in each cell in the first row shows the number of fruitbodies analysed by culturing.

Table S6. Bacterial orders found in mushrooms and/or in soil using high throughput sequencing (HTS).

**Fruitbodies (HTS)**	**Soil (HTS)**
Acidimicrobiales	Acidimicrobiales
Acidobacteriales	Acidobacteriales
Actinomycetales	Actinomycetales
Bacillales	Bacillales
Bdellovibrionales	Bdellovibrionales
Burkholderiales	Burkholderiales
Caulobacterales	Caulobacterales
Chlamydiales	Chlamydiales
Chthoniobacterales	Chthoniobacterales
Clostridiales	Clostridiales
Corynebacteriales	Corynebacteriales
Enterobacteriales	Enterobacteriales
Entomoplasmatales	Entomoplasmatales
Frankiales	Frankiales
Gaiellales	Gaiellales
Gemmatimonadales	Gemmatimonadales
Holophagales	Holophagales
Lactobacillales	Lactobacillales
Legionellales	Legionellales
Methylacidiphilales	Methylacidiphilales
Micrococcales	Micrococcales
Myxococcales	Myxococcales
Nitrospirales	Nitrospirales
Obscuribacterales	Obscuribacterales
Oligoflexales	Oligoflexales
Opitutales	Opitutales
Planctomycetales	Planctomycetales
Pseudomonadales	Pseudomonadales
Pseudonocardiales	Pseudonocardiales
Rhizobiales	Rhizobiales
Rhodocyclales	Rhodocyclales
Rhodospirillales	Rhodospirillales
Rickettsiales	Rickettsiales
Solibacterales	Solibacterales
Solirubrobacterales	Solirubrobacterales
Sphingobacteriales	Sphingobacteriales
Sphingomonadales	Sphingomonadales
Streptomycetales	Streptomycetales
Syntrophobacterales	Syntrophobacterales
Tepidisphaerales	Tepidisphaerales
TM6	TM6
unclassif. Acidobacteriia	unclassif. Acidobacteriia
unclassif. Alphaproteobacteria	unclassif. Alphaproteobacteria
unclassif. Bacteria	unclassif. Bacteria
unclassif. Betaproteobacteria	unclassif. Betaproteobacteria
unclassif. Chloroflexi	unclassif. Chloroflexi
unclassif. Cyanobacteria	unclassif. Cyanobacteria
unclassif. Deltaproteobacteria	unclassif. Deltaproteobacteria
unclassif. Firmicutes	unclassif. Firmicutes
unclassif. Gammaproteobacteria	unclassif. Gammaproteobacteria

unclassif. Parcubacteria	unclassif. Parcubacteria
unclassif. Phycisphaerae	unclassif. Phycisphaerae
unclassif. Proteobacteria	unclassif. Proteobacteria
unclassif. Verrucomicrobia	unclassif. Verrucomicrobia
WD272	WD272
Xanthomonadales	Xanthomonadales
Aeromonadales*	Catenulisporales*
Alteromonadales*	Chlorobiales*
Bacteroidales*	Ktedonobacterales*
Bifidobacteriales*	Pedosphaerales*
Campylobacterales*	Phycisphaerales*
Candidate division OP3*	Selenomonadales*
Candidatus Saccharibacteria*	Streptosporangiales*
Chromatiales*	unclassif. Acidobacteria*(2)
Cytophagales*	unclassif. Holophage*(1)
Deinococcales*	unclassif. Clostridia*(1)
Flavobacteriales*	unclassif. Elusimicrobia*(1)
Hydrogenophilales*	unclassif. Ktedonobacteria*(1)
Methylococcales*	unclassif. Omnitrophica*(1)
Neisseriales*	unclassif. Planctomycetacia*(1)
Nitrosomonadales*	unclassif. Planctomycetes*(3)
Oceanospirillales*	
Pasteurellales*	
Propionibacteriales*	
Rhodobacterales*	
Rubrobacterales*	
Thermales*	
Thermoanaerobacterales*	
unclassif. Actinobacteria*(1)	
Verrucomicrobiales*	

*asterisk marks unique orders for fruitbodies or for soil.

The number of unclassified orders are shown in parentheses.
