# Supplementary material for: Bacterial Communities in Boreal Forest Mushrooms Are Shaped Both by Soil Parameters and Host Identity
Source: Front Microbiol. 2017 May 10;8:836. doi: 10.3389/fmicb.2017.00836 (PMC5423949; doi:10.3389/fmicb.2017.00836)
Supplement: Supplementary file 5 [file Data_Sheet_5.pdf]

Table S9. Effect of soil parameters, fungal taxonomy, soil type, habitat type and site on bacterial community composition in fungal fruitbodies as revealed by PERMANOVA using single-factor analysis. P-value is based on 999 permutations. Data matrix based on rarefied, Hellinger transformed HTS read numbers.

	Df	SS	MS	F	R^2	R^2adj	p
**Soil pH**	1	1.9065	1.90646	6.2427	0.07690	0.0620	0.001
**Fungal order**	3	3.3701	1.12338	3.6785	0.13594	0.0927	0.001
**Soil δ15N**	1	0.7247	0.72472	2.3731	0.02923	0.0136	0.006
**Fungal genus**	4	2.2984	0.57461	1.8816	0.09271	0.0312	0.001
**Soil C:N (Observed/measured)**	1	0.2580	0.25796	0.8422	0.01041	-0.0056	0.617
**Soil C:P**	1	0.4139	0.41393	1.3646	0.01670	0.0008	0.132
**Habitat type**	2	0.6160	0.30798	1.0088	0.02485	-0.0071	0.429
**Soil C content**	1	0.2125	0.21246	0.6917	0.00857	-0.0074	0.832
**Soil Ca content**	1	0.4124	0.41237	1.3593	0.01663	0.0008	0.129
**Soil K content**	1	0.3245	0.32453	1.0639	0.01309	-0.0028	0.357
**Soil Mg content**	1	0.3464	0.34639	1.1372	0.01397	-0.0019	0.296
**Soil N content**	1	0.2119	0.21192	0.6900	0.00855	-0.0074	0.846
**Soil organic matter**	1	0.3352	0.33522	1.0997	0.01352	-0.0024	0.309
**Soil P content**	1	0.3219	0.32187	1.0551	0.01298	-0.0029	0.372
**Soil N:P**	1	0.3957	0.39569	1.3030	0.01596	8.83871e-05	0.158
**Site**	9	3.9424	0.43804	1.5708	0.15903	0.0189	0.001
**Soil type**	3	0.8486	0.28286	0.9222	0.03423	-0.0141	0.600
**Soil C:N (Calculated)**	1	0.2581	0.25809	0.8426	0.01041	-0.0056	0.638
**Total**	63	24.7907			1.00000		

Table S10. Results of PERMANOVA analysis of bacterial community composition revealed from presence/absence dataset (aggregated data of culturing and HTS) in fungal fruitbodies in relation to biotic and abiotic factors.

	Df	SS	MS	F.Model	R^2	R^2adj	p
**Soil pH**	1	1.6369	1.63695	6.4334	0.07374	0.0603	0.001
**Fungal order**	3	2.9789	0.99298	3.9025	0.13419	0.0954	0.001
**Soil d15N**	1	0.5277	0.52765	2.0737	0.02377	0.0096	0.038
**Fungal genus**	4	1.5352	0.38381	1.5084	0.06916	0.0127	0.027
**Residuals**	61	15.5212	0.25445		0.69915	-1.3399	
**Total**	70	22.2000			1	1	

Table S11. Results of PERMANOVA analysis of bacterial community composition based on rarefied, Hellinger transformed HTS read numbers in fungal fruitbodies in relation to biotic and abiotic factors.

	**Df**	**SS**	**MS**	**F.Model**	**R^2**	**R^2adj**	**p**
**Fungal order**	3	3.9343	1.31142	4.2943	0.15870	0.1166	0.001
**Fungal genus**	4	2.8641	0.71601	2.3446	0.11553	0.0556	0.001
**Soil pH**	1	0.9715	0.97154	3.1813	0.03919	0.0237	0.001
**Soil d15N**	1	0.5299	0.52990	1.7352	0.02137	0.0056	0.038
**Residuals**	54	16.4910	0.30539		0.66521	-1.3435	
**Total**	63	24.7907			1	1	
